# Features and trends of marine heat waves and marine cold spells along the Western Iberian Coast from four decades of satellite data

**DOI:** 10.1038/s41598-025-31504-1

**Published:** 2025-12-24

**Authors:** Beatriz Biguino, Ivan D. Haigh, João Miguel Dias, Ana C. Brito

**Affiliations:** 1 Marine and Environmental Sciences Centre (MARE) / Aquatic Research Network (ARNET), Faculdade de Ciências, Universidade de Lisboa, Lisboa, 1749-016 Portugal; 2 School of Ocean and Earth Science, National Oceanography Centre, University of Southampton, Waterfront Campus, European Way, Southampton, SO14 3ZH UK; 3 Centre for Environmental and Marine Studies (CESAM), Departamento de Física, Universidade de Aveiro, Aveiro, 3810-193 Portugal; 4 Departamento de Biologia, Faculdade de Ciências, Universidade de Lisboa, Lisboa, 1749-016 Portugal; 5 National Center for Integrated Coastal Research and Department of Civil, Environmental and Construction Engineering , University of Central Florida, Orlando, FL, USA

**Keywords:** Estuarine systems, Climate change, Portugal, Galicia, Upwelling, Climate sciences, Environmental sciences, Ocean sciences

## Abstract

Marine Heat Waves (MHWs) have been the focus of numerous studies due to the dramatic consequences they can have on coastal systems. However, Marine Cold Spells (MCSs) can also have harmful effects on the environment while play an important role in the context of global warming. Yet, there is lack of information on the physical attributes and long-term changes of MCSs. This study aims to investigate and compare the features and patterns of both MHWs and MCSs along the Western Iberian Coast (WIC) and its estuaries using satellite-derived Sea Surface Temperatures between 1982 and 2022. Overall, the WIC registered more MCSs than MHWs, although with lower intensities. The coastal region between Minho and Douro Estuaries and Cape São Vicente were favorable for MCSs development. The coastal regions of Minho through Aveiro and the south coast of Portugal registered MCSs and MHWs with the highest average and maximum intensities. No significant trends were observed for MHWs and MCSs features throughout the WIC. MHWs (MCSs) events were found to have been increasing (decreasing) offshore. However, an increase was registered near the coastline for MCS (0.06 events/year). Increased seasonal upwelling could be contributing to mask the development of MHWs and enhance MCSs.

**Supplementary Information:**

The online version contains supplementary material available at 10.1038/s41598-025-31504-1.

## Introduction

Climate change is dramatically shaping marine systems. One of the clearest signs of this is the worldwide increase of global ocean temperatures and the consequent effects it has on ecosystems^1^. These include sea level rise, ocean acidification (driven by higher CO_2_ absorption), increased density stratification and loss of oxygen^1,2^. Along with a long-term warming signal, an increase in the frequency and intensity of extreme temperature-related events has also been observed^3^. Marine systems are strongly influenced by extreme climatic events, such as storms, floods and heat waves. These derive from complex physical processes interconnected in the climate system but are often enhanced by high water temperatures^3,4^. For example, Tropical Cyclone Yaku, the first such storm to hit Peru in decades, and the intense rainfall felt on the region were fed by unusually warm waters^4^. This increase in frequency and intensity of weather extremes is important, as it can directly affect ocean health, change ecosystem services and disrupt economic activities, altering the normal living of coastal populations. Marine systems are central in most people’s lives, culturally, socially and economically^3^. Thus, understanding temperature-related extreme weather events and their effects in ecosystems is crucial.

Recent decades have seen many high-impact MHWs^5^. MHWs are defined by periods of abnormally high ocean temperatures relative to the local average seasonal temperature and tend to be less understood than their terrestrial counterparts^3,6^. These extreme warm sea surface temperatures can persist for days to months and extend to thousands of kilometers, likely causing a change in marine ecosystem structure and functioning^3,7^. Strong MHWs in some subtropical regions are strongly linked to ENSO and atmospheric anomalies (persistent atmospheric high-pressure systems and anomalously weak wind speeds, along with increased insolation and reduced ocean heat losses)^8^. MCSs are the opposite of MHW, and are prolonged events characterized by abnormally low water temperatures, often developed due to wind-induced ocean processes^9^. MCSs can also have critical negative impacts on marine ecosystem structure (e.g., mass mortalities, range shifts, marine habitat loss) but may benefit marine ecosystem recovery when damaged by MHWs and buffer the impacts of heat stress during those type of events^9,10^. MCSs have received less global attention than MHWs but their study is essential to characterize ocean temperature extremes and understand marine ecosystems thermal bounds evolution^9^. These events affect open-ocean environments but also coastal and transitional systems, with consequences yet to be fully identified for ecosystems and populations.

Worldwide MHWs have been increasing in their incidence, duration, and intensity in response to greenhouse warming^5,11,12^. These were the patterns found regionally in the Mediterranean Sea (between 1982 and 2021;^13^, coastal New Zealand^14^, the Black Sea^15^, the European coastal Northeast Atlantic^16^ and the Northern Arabian Sea^17^. Continuous global warming is expected to intensify these changes in the future^7^. Still, the Mediterranean Sea has been one of the regions most susceptible to these changes due to its rapid response to climate change^18^. In opposition, MCS duration and intensity have been declining throughout the global ocean, except for the Southern Ocean where an increase has been observed for the past 15 years^9^. One of the largest MCSs identified in the satellite records affected the North Atlantic between 2013 and 2016 and it was named “cold blob”, in opposition to “the blob”, a well-known Northeast Pacific MHW that appeared between 2014 and 2016. The Northeast Pacific Blob lasted 711 days and had a maximum intensity of 2.56 ℃, being classified a Category III MHW^5^.

Despite the recent progress, current knowledge about past occurrences and the future progression of this type of events is limited^7^. Moreover, the changes reported for MHWs and MCSs are not regionally uniform^16^. Currently, it is still unclear if coastal areas, including estuarine systems, follow open-ocean trends^16^, which constitute relevant knowledge gaps. High-resolution coastal studies focusing on both MHWs and MCSs are needed to bridge the gap and allow the development of appropriate regional mitigation and adaptation plans, namely along the Western Iberian Coast (WIC), where information on this subject is very scarce.

Therefore, the main aim of this study was to identify features and patterns of past events of both MHWs and MCSs along the WIC in a very high-resolution approach (5 km). Three objectives were established: *(i)* identify past events and classify them according to their maximum intensity, determining the most favorable regions to the occurrence of MHWs and MCSs; *(ii)* evaluate the average patterns of the last 41 years regarding the duration and average, maximum and cumulative intensity of MHWs and MCSs; and *(iii)* investigate the trends in the annual frequency of past MHWs and MCSs events and their characteristics (duration, first day of the event and average, maximum and cumulative intensity). Special focus was also given to the estuarine systems of the WIC. Here, the contrast between MHWs and MCSs is assessed through a long-term portrait of these events and their physical attributes in an integrated and continuous view of the WIC and its estuaries, with a seasonal evaluation of the observed patterns. This will be a relevant contribution to the definition of adaptation and mitigation measures for Portuguese and Spanish marine environments.

The paper is structured in six sections as follows: Sect. 2 introduces the study site and Sect. 3 focuses on the data and methods used in this study. Section 4 presents the results in three subsections, according to objectives proposed. These are then discussed in Sect. 5. Lastly, the main conclusions of this work are drawn in Sect. 6.

## Study sites: Western Iberian Coast and its estuarine systems

**Fig. 1:**
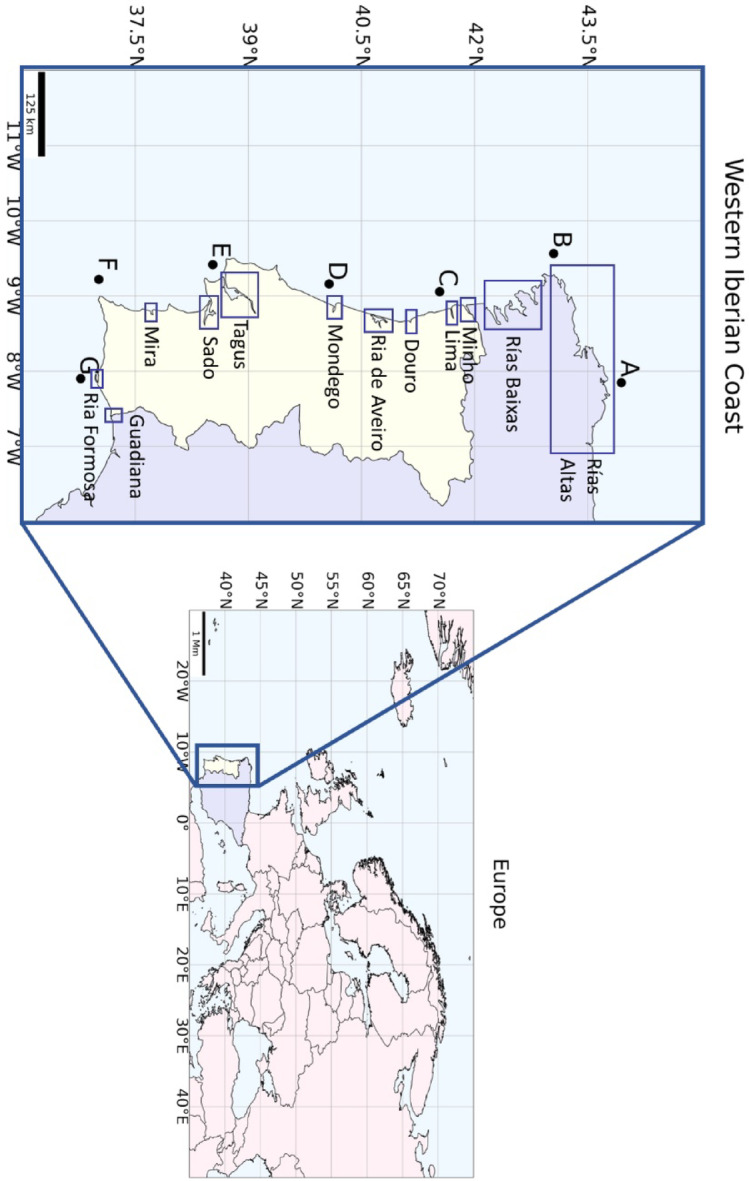
Framing of the Western Iberian Coast within Europe and location of the main estuarine systems of Portugal and Galicia (blue squares). A to G represent the locations of coastal points where MHWs/MCSs were studied.

At the confluence between the Atlantic Ocean and the southwestern corner of Europe, we find the Iberian Peninsula (Fig. 1). The Western Iberian Coast (WIC) connects mainland Portugal with the region of Galicia (Spain), and it is shaped by several estuaries and capes that mark its morphology, influence circulation patterns, and enhance the variability of the physical and biological features of the coast^19^. Some of the main estuarine systems of the region include: Rías Altas, Rías Baixas, Minho Estuary, Lima Estuary, Douro Estuary, Ria de Aveiro, Mondego Estuary, Tagus Estuary, Sado Estuary, Mira Estuary, Ria Formosa and Guadiana Estuary (Fig. 1). The most relevant rias from Rias Altas are La Coruña, Betanzos and Ferrol, which form a distinct group^20^. Rías Baixas take in four rias, Muros-Noia, Arousa, Pontevedra and Vigo, all with 8–12 km extension in their external part and a central axis lying in a SW–NE direction^21^. Tagus and Sado are the largest Portuguese systems, with areas of 320 and 180 km^2^, respectively^22^. Information on the main geomorphologic and hydrologic characteristics of the Portuguese and Galician estuarine systems can be found in^22] and [20^, respectively.

A Mediterranean type of weather characterizes the region with warm/hot summers^23^. The area is framed in the Eastern North Atlantic Upwelling System, similarly to other eastern boundary current systems, such as California^19^. The region is influenced by upwelling events, mainly during spring and summer. The predominance of northerly winds specially during these seasons, leads to the emergence of cooler and nutrient-rich subsurface waters, that can reach up to 200 km offshore and are of high relevance for national fisheries^24,25^. In opposition, during winter, westerly and southerly winds prevail and form the Iberian Poleward Current^24,26^.

**Table 1:** Location and coordinates of the 7 coastal stations (A-G) considered in this study.

Station	Location	Latitude (° *N*)	Longitude (° W)
A	Ortigueira Coast	43.947	007.849
B	Finisterre Coast	43.050	009.562
C	Lima Estuary Coast	41.537	009.056
D	Mondego Estuary Coast	40.070	009.160
E	Tagus Estuary Coast	38.527	009.415
F	Cape São Vicente	37.016	009.220
G	Ria Formosa Coast	36.775	007.902

## Data and methods

Two subsections focusing respectively on the data used and the methods considered in the analysis are presented below.

### Data

Satellite-derived Sea Surface Temperatures (SST) were used in the present study. The SST product analyzed was produced by the ESA Climate Change Initiative (CCI) and the SST CCI project^27,28^ and has daily estimates of global SSTs with a spatial resolution of 0.05 ° (≈ 5 km). This is a level-4 dataset with data gathered from multiple satellite sensors. The time span chosen for this study covered the period from the 1st of January 1982 to the 31st of December 2022.

The SST satellite data was previously validated for the coastal ocean of the WIC through the comparison with concomitant in situ observations gathered from coastal monitoring buoys^29^. The lack of long-term SST series of in situ observations made it impossible to validate the product for every estuarine system, but very reasonable results were obtained for the Tagus and Sado estuaries in a matchup assessment conducted considering several sampling stations (Tagus Estuary: *R*^2^ > 0.89, − 0.13 > *BIAS*>− 0.50, *RMSE* < 0.92; Sado Estuary: *R*^2^ > 0.62, 0.07 > *BIAS*>− 2.03, *RMSE* < 3.44). To validate the satellite data for the Tagus Estuary, in situ observations were retrieved from CoastNet Research Infrastructure (http://coastnet.pt). Aquasado Project (MAR-02.01.91‐FEAMP‐0051) provided in situ observations for the Sado Estuary. Additionally, the spatial resolution of the satellite product made it difficult to look inside the smaller estuarine systems and rely on the most detailed results.

### Methods

Two subsections focusing on the methods for the identification and classification of MHWs and MCSs and for the average patterns and trend analysis are described below.

#### Identification and classification

Hobday et al.^3^ proposed a definition to identify MHWs that has been adopted worldwide by the scientific community and currently allows a coherent and comparable framework for the study of these type of events. A MHW is therefore a period of at least five consecutive days with water temperatures above the 90th percentile threshold of the long-term mean seasonal climatology (static). After their identification, it is also possible to classify these events according to their intensity. Considering multiples of the local difference between the climatological mean and the climatological 90th percentile, four categories were proposed by^5^ as follows: moderate (1–2×, Category I), strong (2–3×, Category II), severe (3–4×, Category III) and extreme (> 4×, Category IV)^5^. Thus, as an example, if the maximum water temperature observed during the MHW period is 2.5× the difference between the climatological mean and the climatological 90th percentile, we are facing a Category II MHW. The definitions for identifying and classifying MHWs were also adapted for MCSs as the inverse of the MHWs – cold water temperature anomalies below the 10th percentile for more than five continuous days^9^. A python module called *marineHeatWaves* (version 0.28) created and provided by Eric C. J. Oliver was used as a basis for the identification of events and their characteristics (*detect* function)^30^.

The identification and classification of MHWs and MCSs was performed for 7 strategically chosen points (A-G), equally distributed along the WIC and placed at 20 km from the coast (Fig. 1; Table 1), through the analysis of SST time series extracted from each point. The choice of these coastal points was based on the pre-established oceanographic relevance of the areas for the study of MHWs and MCSs (e.g., their higher or lower propensity to upwelling development or water temperature increases;^29^. Additionally, the points were chosen to represent the coastal region of some estuarine systems and relevant capes. This point-based comparison aimed to classify the number of events observed in these strategically selected areas and to study the characteristics of particular events. Given the high spatial resolution of the satellite product used (5 km) and the aim of this phase of the analysis, selecting an adjacent pixel would not compromise the results of this evaluation. Although some variability was observed, the chosen regions still capture the overall behavior of both the focal pixel and its neighboring ones. This is shown in Supplementary Table S1, where the variance and the coefficient of variation of the number of MHWs and MCSs are presented for the 3 × 3 pixel frame of each location. Table 1 shows the location and coordinates of the stations. These non-gap time series were considered for identification of MHWs/MCSs events and for their classification. The period between 1982 and 2022 was considered the reference climatology.

The events identified were also studied regarding the season of the year in which they started. For this purpose, the following was considered: winter - January, February and March; spring - April, May and June; summer – July, August and September; autumn – October, November and December. The most favorable regions for the occurrence of MHWs/MCSs were identified considering the total number of events that occurred throughout the WIC between 1982 and 2022. The regions with higher incidence of past occurrences were identified as most propense for the occurrence of these type of events.

#### Average patterns and trend analysis

MHWs/MCSs statistics were calculated using the blockAverage function from the *marineHeatWaves* python module, where the event’s physical attributes were averaged over annual blocks. From this, information was retrieved to produce the average and trend maps. 41-year averages were calculated for the following features: duration and average, maximum and cumulative intensity of MHWs/MCSs. The cumulative intensity refers to the intensity cumulated over the duration of the event (℃ x days). The trend analysis was conducted considering: annual frequency of past MHWs/MCSs events, average duration, first day of the event and the annual mean average, maximum and cumulative intensity. This trend assessment was also repeated considering the seasons of the year, i.e., the changes per year were studied considering the data from each season separately. Regarding the first day of the events, the averaging of annual blocks was not applied and only the day of the first event of each year was considered. Linear models were applied to the SST time series and complemented with a statistical assessment, including linear regression and the significance of the trend (*p-value*, significant if lower than 0.05). Only significant trends were colored in the trend maps. All these analyses followed a pixel-by-pixel approach. A big focus was given to estuarine systems, as key environments in coastal management.

## Results

The results obtained are presented in the following subsections according to the three study objectives.

### Identification and classification

**Fig. 2:**
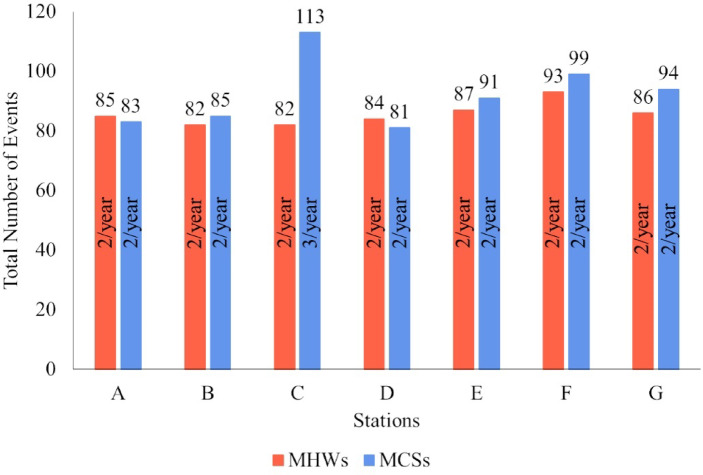
Total number of Marine Heat Waves (MHWs) and Marine Cold Spells (MCSs) identified in 7 locations of the Western Iberian Coast (A-G) between 1982–2022. Inside the bars is the average number of events recorded per year.

The first objective of this study was to identify past MHWs and MCSs events and classify them according to their maximum intensity. Seven coastal locations (identified from A to G in Fig. 1) were considered for this assessment. Figure 2 shows the total number of MHWs and MCSs identified over the 41-year period under analysis and the annual average. The region where the most MHWs were observed was not the region with the lowest number of MCSs. However, the opposite was true: the station where the most MCSs were identified was the region where the fewest MHWs were observed. Of the 7 stations considered, it was near Cape São Vicente (Station F) that the highest number of MHWs (93) was identified. More MCSs than MHWs were identified at each station, except for stations A and D. Also, there is greater spatial variability regarding the occurrence of MCSs than of MHWs. Overall, almost two MHWs and MCSs events were registered per year. Supplementary Figure S1 shows the number of MHWs and MCSs observed each year of the studied period at the 7 locations. The occurrence of both type of events was scattered throughout time and no evident patterns were observed. Still, there appears to have been a decrease in the number and MCSs over time in region A, and an increase in the number of MHWs in region G. Supplementary Figure S2 shows the total number of MHWs and MCSs obtained for the 7 studied coastal locations of the WIC according to the season of the year in which they started. Overall, summer was the most prominent season for the occurrence of both MHWs and MCSs. As for MHWs, the seasons with the highest number of events were spring/summer and summer/autumn, interspersed throughout the WIC. There were fewer events starting in winter. MCSs began predominantly between summer and autumn in the northernmost regions of WIC (stations A and B). On the west coast of WIC, the predominance fell in spring and summer (stations C, D and E), while on the south coast there was a more equal distribution throughout the year.

**Fig. 3:**
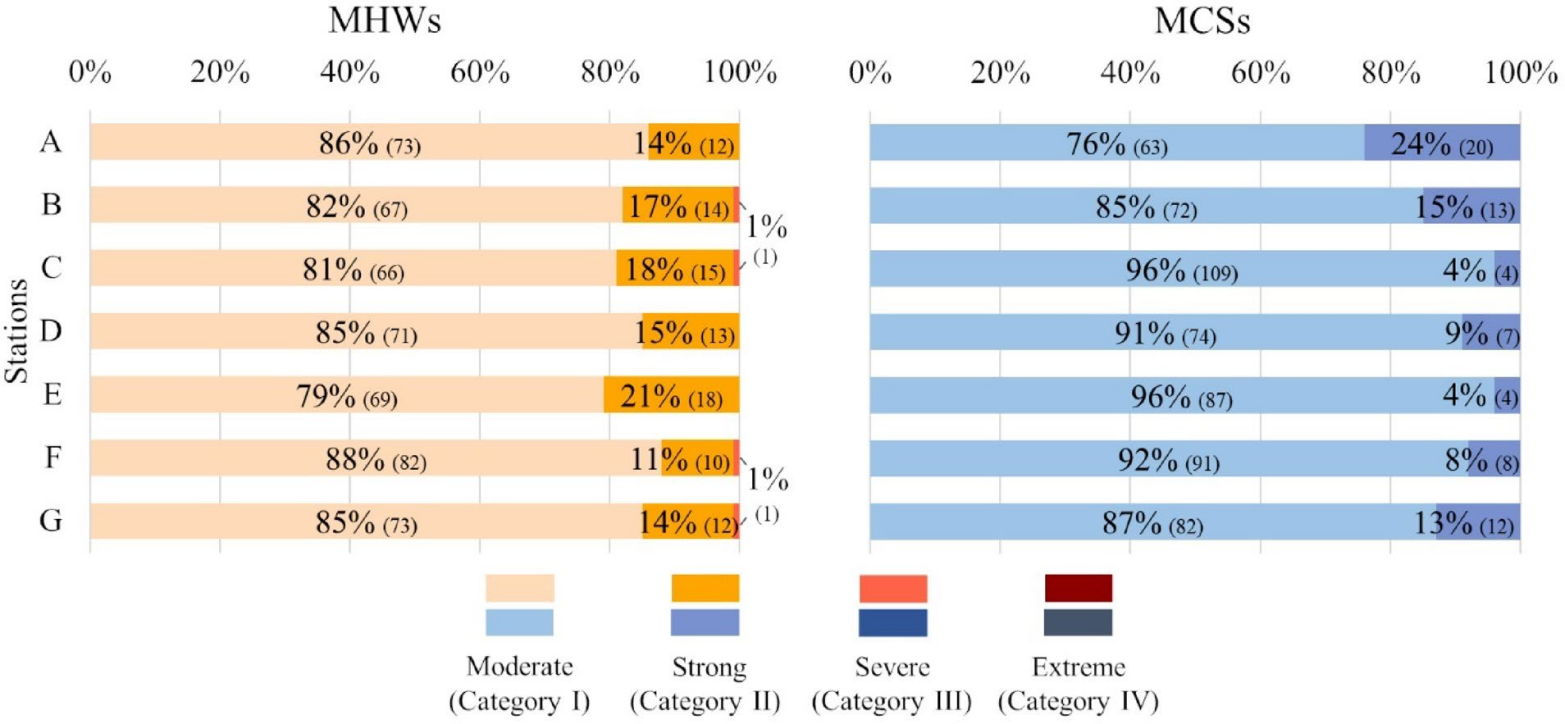
Classification of the identified Marine Heat Waves (MHWs) and Marine Cold Spells (MCSs) according to the four categories established by^5^. The percentage is relative to the total number of events observed in each station. Inside the parentheses is the total number of events identified for each category.

Figure 3 presents the classification of the identified events according to the four categories established by^5^. One Severe MHW (Category III) was observed in stations B, C, F and G. The remaining locations did not record events of such intensity. The mouth of the Tagus Estuary (station E) had the highest incidence of Strong events (Category II; 21%) despite not having recorded the highest total number of MHWs. Despite the overall greater number of MCSs, these events never exceeded Category II intensities. It was in the north of Galicia (station A) that a higher incidence of category II MCS was observed (24%), this being the second location with fewer events identified. The coastal region near Lima Estuary (Station C) suffered the greatest occurrence of MCSs but these were overall moderate (96%). There were no Extreme events (Category IV) for either MHWs or MCSs.

Table 2 presents the physical attributes of the highest maximum intensity events that were observed between 1982 and 2022 at the 7 sampling stations. The events detected were spatially restricted. There was only one case in which the maximum intensity event was the same in more than one station, the MCS from stations E and F. This means that this event extended with similar characteristics at least from the Tagus mouth to Cape São Vicente. The maximum intensity of this event was reached later in Cape São Vicente than near Tagus. The MHW at station G could also have been a delayed manifestation of the MHW observed in region D. The highest intensity MHWs were observed in the different regions between spring and summer (June-September), while MCSs were observed between summer and autumn (August-October). Overall, the higher intensity MHWs reached higher intensities than the equivalent MCSs. The 2011 Cape São Vicente MHW was the event that recorded the highest maximum intensity (4.88 ℃). It lasted 61 days and consequently, was rated as Severe. In these coastal regions, all the greatest intensity MHWs were recorded prior to 2011. On the other hand, there were maximum intensity records reached in 2019 for the MCS (region B), much more recently. It was in region D that the most intense MCS was observed (− 4.04 ℃ in 2002).

A Severe MHW was also detected in the inner region of the Tagus Estuary, which had a maximum and average intensity of 3.18 ℃ and 1.94 ℃, respectively, and lasted 43 days between 28 FEB 1997 and 11 APR1997. This information does not invalidate the fact that other systems may have also suffered events of similar classification, even though the Tagus appears to be one of the most susceptible.

**Table 2:** Main features of the MHW and MCS with the highest maximum intensity (I Max) observed in each one of the 7 locations of the Western Iberian Coast (A-G). * Same event. Bold highlights the higher values.

Station	Classification	Duration (days)	I Max (℃)	Start and End Date	Peak Date	Season
Marine Heat Waves						
A	Strong (Category II)	19	3.14	12–30 JUN 2008	22 JUN 2008	Early Summer
B	Strong (Category II)	7	4.05	24–30 AUG 1999	27 AUG 1999	Summer
C	Strong (Category II)	31	4.53	26 JUL − 25 AUG 2004	22 AUG 2004	Summer
D	Strong (Category II)	23	4.72	29 MAY − 20 JUN 2006	12 JUN 2006	Spring/ Early Summer
E	Strong (Category II)	23	4.11	27 JUL − 18 AUG 1989	07 AUG 1989	Summer
F	Severe (Category III)	**61**	**4.88**	13 APR − 12 JUN 2011	05 JUN 2011	Spring
G	Strong (Category II)	22	4.40	28 AUG − 18 SEP 2006	07 SEP 2006	Late Summer
Marine Cold Spells						
A	Strong (Category II)	20	− 3.96	03–22 SEP 2007	07 SEP 2007	Late Summer
B	Strong (Category II)	18	− 3.85	01–18 SEP 2019	06 SEP 2019	Late Summer
C	Moderate (Category I)	15	− 3.46	04–18 OCT 1996	10 OCT 1996	Autumn
D	Strong (Category II)	14	**-4.04**	30 JUL − 12 AUG 2002	01 AUG 2002	Summer
E	Strong (Category II)*	26	− 3.23	10 SEP − 05 OCT 2017	21 SEP 2017	Late Summer/Autumn
F	Strong (Category II)*	21	− 3.62	09–29 SEP 2017	23 SEP 2017	Late Summer/Autumn
G	Strong (Category II)	**42**	− 3.70	22 SEP − 02 NOV 1993	01 OCT 1993	Autumn

**Fig. 4:**
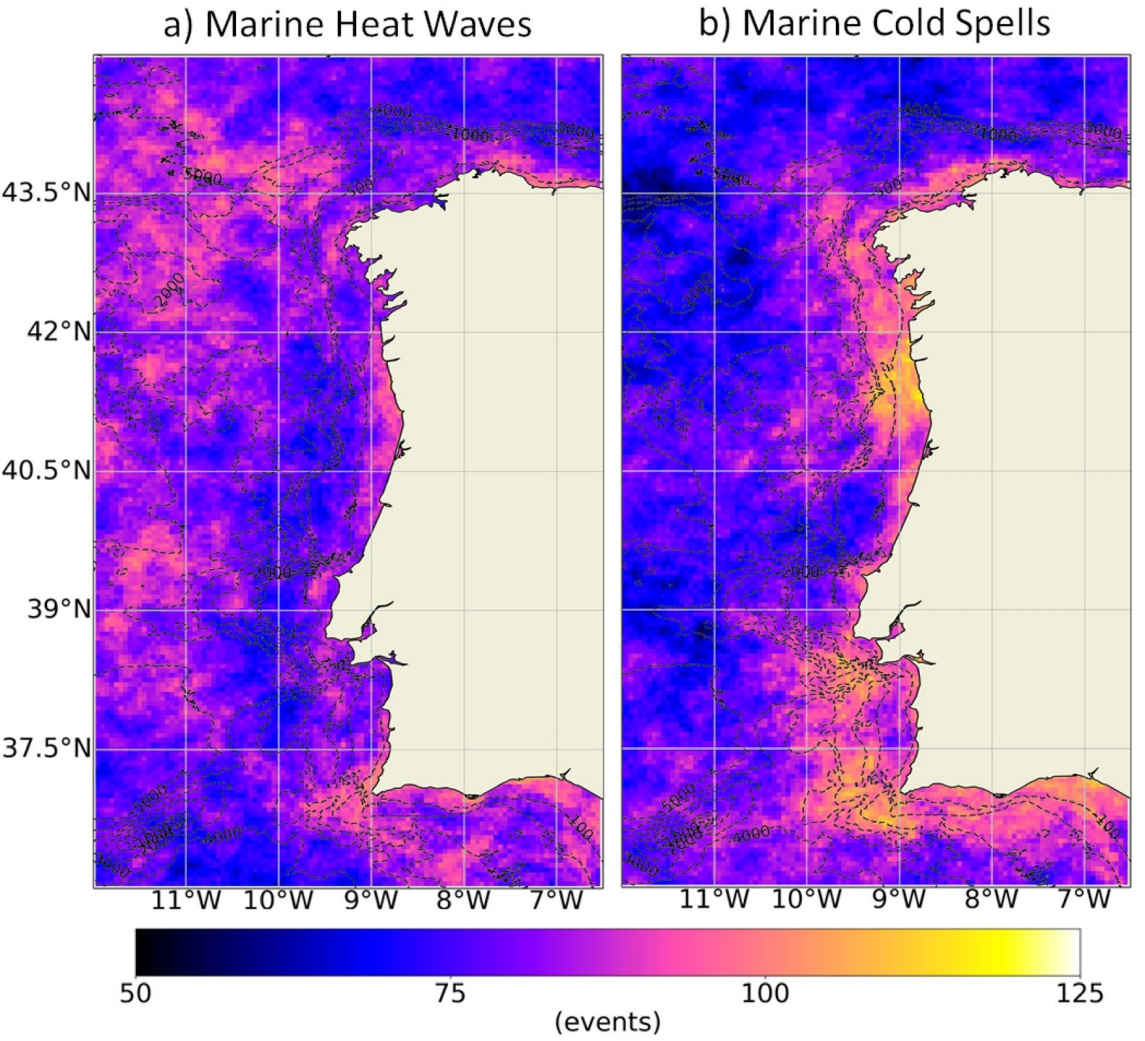
Total number of MHWs (**a**) and MCSs (**b**) that occurred between 1982 and 2022 along the Western Iberian Coast, with reference to the maximum (Max), minimum (Min) and average of the values obtained. Results obtained using the satellite-derived Sea Surface Temperature data from ESA CCI project. Bathymetry data source: GEBCO Compilation Group (2022) GEBCO 2022 Grid (doi: 10.5285/e0f0bb80-ab44-2739-e053-6c86abc0289c).

To identify the most favorable regions for the occurrence of MHWs and MCSs events, integrating the WIC and its main estuarine systems, Fig. 4 maps the total number of events that occurred throughout the region between 1982 and 2022. The spatial patterns derived from this assessment allow us to identify the regions most and least likely to experience these types of events, thereby adding a broader perspective that strengthens and further supports the robustness of the point-based comparison presented in Fig. 2.

**Fig. 5:**
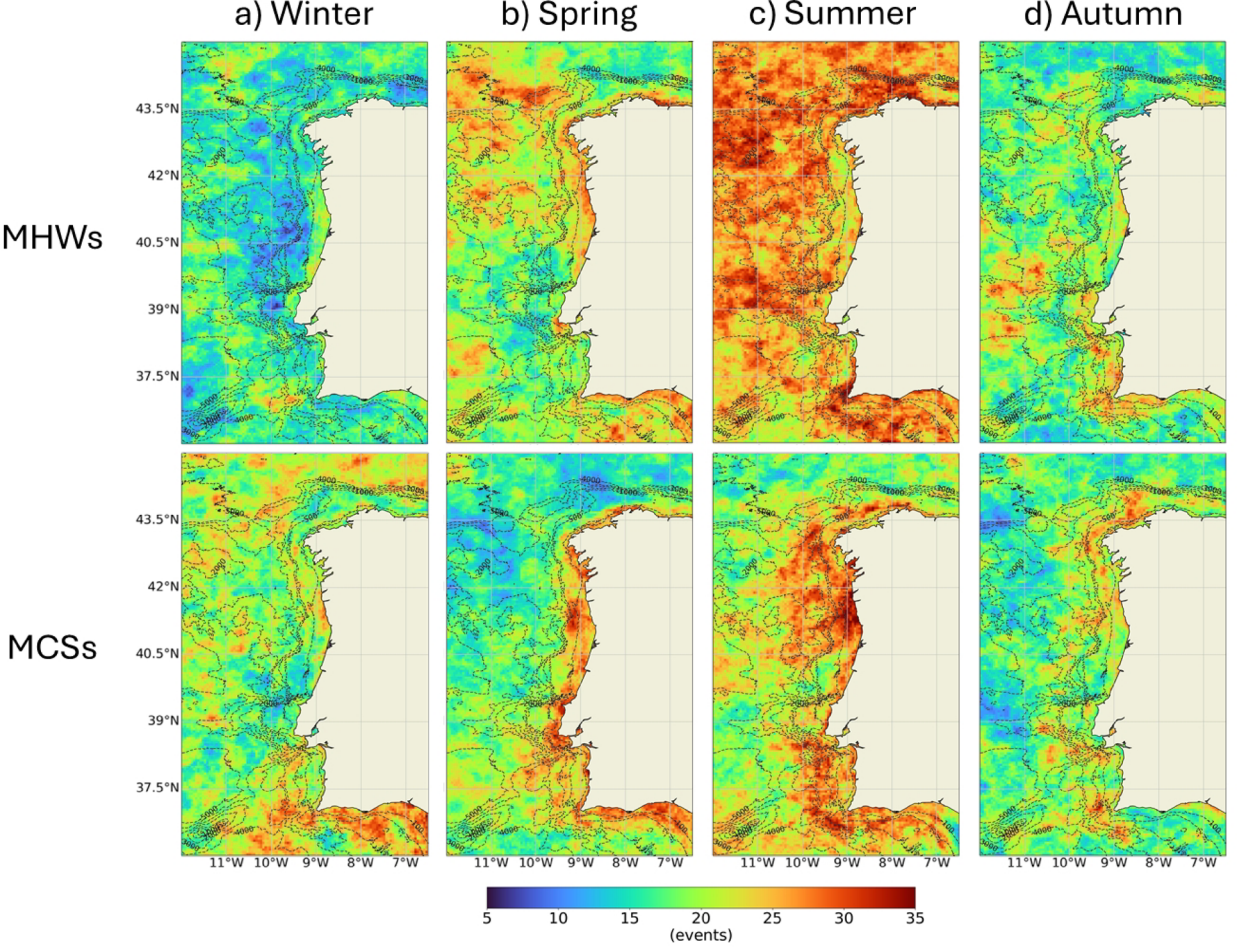
Total number of Marine Heat Waves (MHWs, above) and Marine Cold Spells (MCSs, bellow) that occurred between 1982 and 2022 along the Western Iberian Coast during winter (**a**), spring (**b**), summer (**c**) and autumn (**d**) months. Results obtained using the satellite-derived Sea Surface Temperature data from ESA CCI project. Bathymetry data source: GEBCO Compilation Group (2022) GEBCO 2022 Grid (doi: 10.5285/e0f0bb80-ab44-2739-e053-6c86abc0289c).

Near shore, a greater incidence of MHW was observed along the eastern coast of Rías Altas, the coastal region between Lima Estuary and Ria de Aveiro and the southern Portuguese coastal margin, with a great focus on Cape São Vicente. Similarly, areas with higher incidence of MHWs were also observed offshore. This means that the WIC coast was not exceptionally favorable for the occurrence of these events. In opposition, there was a clear higher incidence of MCSs near the coast than offshore. Between Minho and Douro Estuaries and near Espichel and Cape São Vicente, there appear to have been more favorable conditions for the occurrence of MCSs. Rías Baixas and the Sado Estuary revealed to be most prone regions for MCSs, when comparing with the adjacent coastal area, condition that was not evident in the other estuarine systems. Over the 41 years, the WIC registered on average a slightly higher number of MCSs (62) than of MHWs (57), corroborating the results of the previous section. Similarly, Fig. 5 presents the same results according to the season of the year and reinforces the higher incidence of both MHWs and MCSs in the summer months. Also, that the incidence of MCSs is much more restricted to the coastal region than that of MHWs and that during winter the Algarve coast is particularly favorable to the occurrence of MCSs.

### Average patterns

**Fig. 6:**
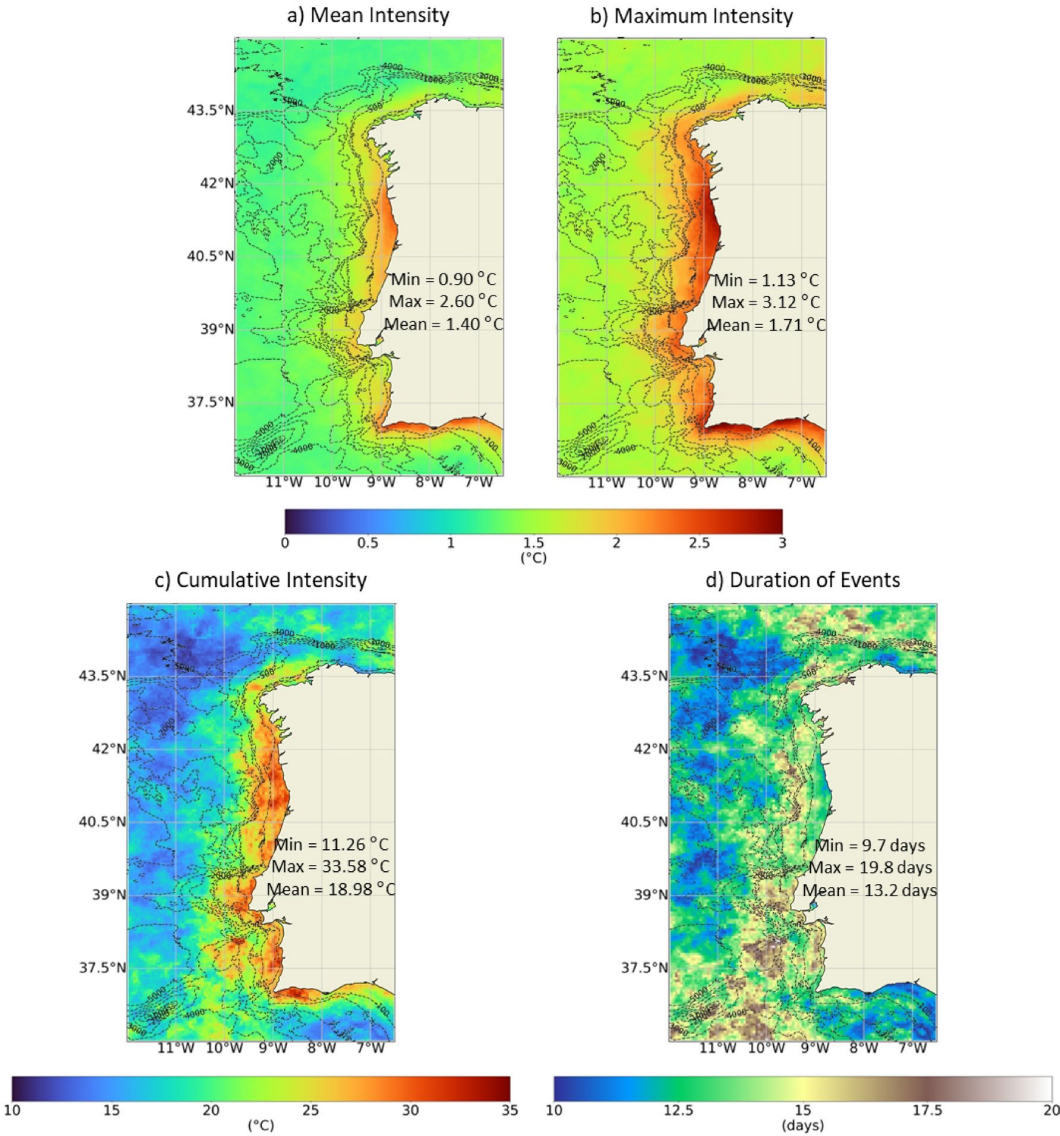
41-year average mapping of the mean (**a**), maximum (**b**) and cumulative (**c**) intensity and duration (**d**) of the MHWs throughout the WIC, with reference to the regional maximum (Max), minimum (Min) and mean of the values obtained. Results obtained using the satellite-derived Sea Surface Temperature data from ESA CCI project. Bathymetry data source: GEBCO Compilation Group (2022) GEBCO 2022 Grid (doi: 10.5285/e0f0bb80-ab44-2739-e053-6c86abc0289c).

The second objective of this study was to evaluate the long-term average MHWs/MCSs patterns between 1982 and 2022. Figure 6 shows the 41-year average duration, mean, maximum and cumulative intensity of the MHWs throughout the WIC. Although a high incidence of MHWs was observed offshore, it was along the coast that the highest mean and maximum intensity events were recorded. Higher centres of mean and maximum intensity were detected along the coast between Minho and Aveiro estuarine systems and along the south coast of Portugal, namely at Cape São Vicente. The regional average of the maximum and mean intensities was 1.71 ℃ and 1.40 ℃, respectively. The highest intensities seem to relate to the bathymetric features of the coast. From a depth of 500 m onward, the intensity of the events decreases. Looking in detail at estuarine systems, the interior of Rías Altas, Rías Baixas and the Sado Estuary were regions of MHW with lower mean intensity and higher duration than the adjacent coastal region. The opposite occurred in the Ria de Aveiro and in the innermost region of the Tagus Estuary. The WIC regions where there was a higher incidence of MHW were, on average, regions where events of shorter duration were recorded (≈ 10 days). The 41-year mapping points to longer events of ≈ 17 days along the coastal area of Southwest Portugal. The cumulative intensity was higher along the entire coast (≈ 27.5–34.5 ℃), following the patterns of the intensity and duration of events.

**Fig. 7:**
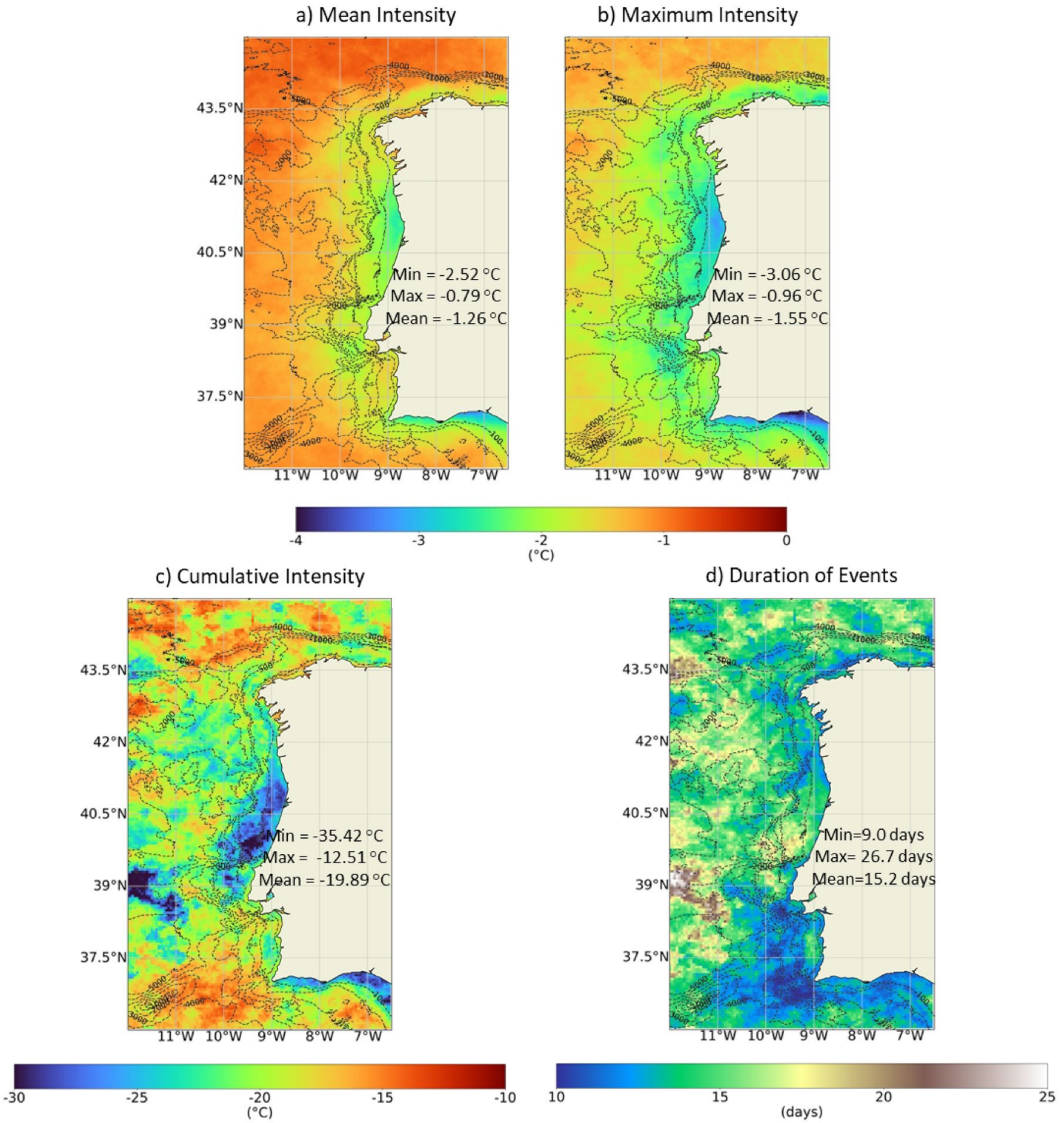
41-year average mapping of the mean (**a**), maximum (**b**) and cumulative (**c**) intensity and duration (**d**) of the MCSs throughout the WIC, with reference to the regional maximum (Max), minimum (Min) and mean of the values obtained. Results obtained using the satellite-derived Sea Surface Temperature data from ESA CCI project. Bathymetry data source: GEBCO Compilation Group (2022) GEBCO 2022 Grid (doi: 10.5285/e0f0bb80-ab44-2739-e053-6c86abc0289c).

Similarly, Fig. 7 shows the 41-year average duration, mean, maximum and cumulative intensity of the MCSs throughout the WIC. It should be noted that higher intensities correspond to lower values, (negative values). The areas of highest mean and maximum intensity were similar for MCSs and MHWs: coastal region between Minho and Aveiro and along the south coast of Portugal. MHWs showed higher intensities than MSCs. As for MHWs, a relation between the bathymetric features of the coast and the intensity patterns of MCSs appears to exist. MCSs lasted longer than MHWs. However, the longest-lasting MCSs were located offshore. In the SW region of Portugal, where the longest MHWs were recorded, the shortest MCSs were identified, (≈ 10 days).

### Trend analysis

**Fig. 8:**
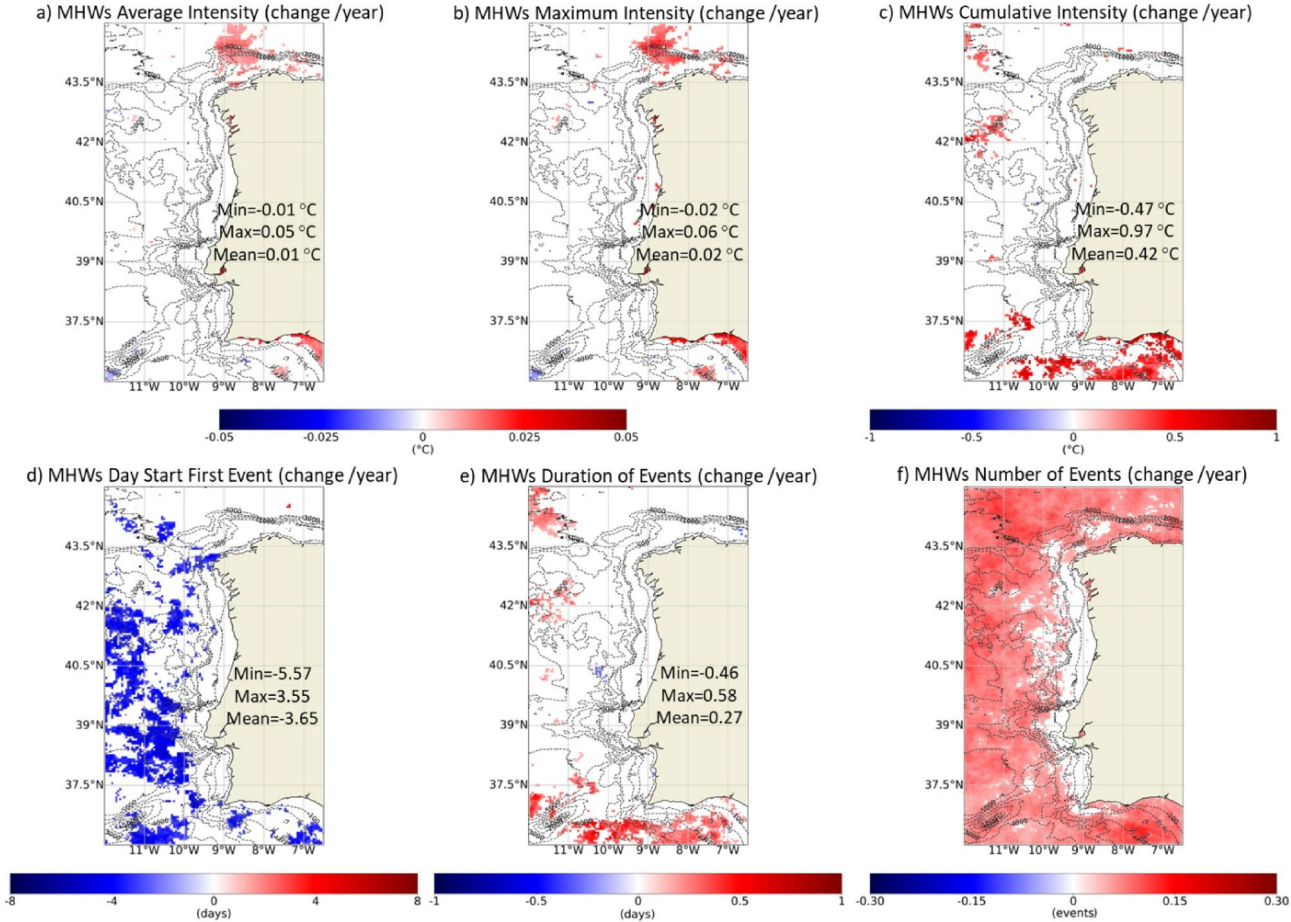
Trends (change/year) of MHWs average (**a**), maximum (**b**) and cumulative intensity (**c**), first day of the event (**d**), duration (**e**) and number of events per year (f) considering the period between 1982 and 2022. Only significant changes are colored (p-value < 0.05). Results obtained using the satellite-derived Sea Surface Temperature data from ESA CCI project. Bathymetry data source: GEBCO Compilation Group (2022) GEBCO 2022 Grid (doi: 10.5285/e0f0bb80-ab44-2739-e053-6c86abc0289c).

The last objective was to perform a trend analysis (change/year) of the annual frequency of past MHWs/MCSs events and their physical attributes. Figure 8 shows the trend results on the average (a), maximum (b) and cumulative intensity (c), first day of the event (d), duration (e) and number of MHWs events per year (f), considering the period between 1982 and 2022. Significant changes of the average, maximum and cumulative intensity of the events were only observed along the continental margin of southern Portugal and the north of Galicia, which was a very localized focus of increased intensity of MHWs. This intensification along the southern coast of Portugal was driven primarily by a marked increase during the summer months (data not shown).

**Fig. 9:**
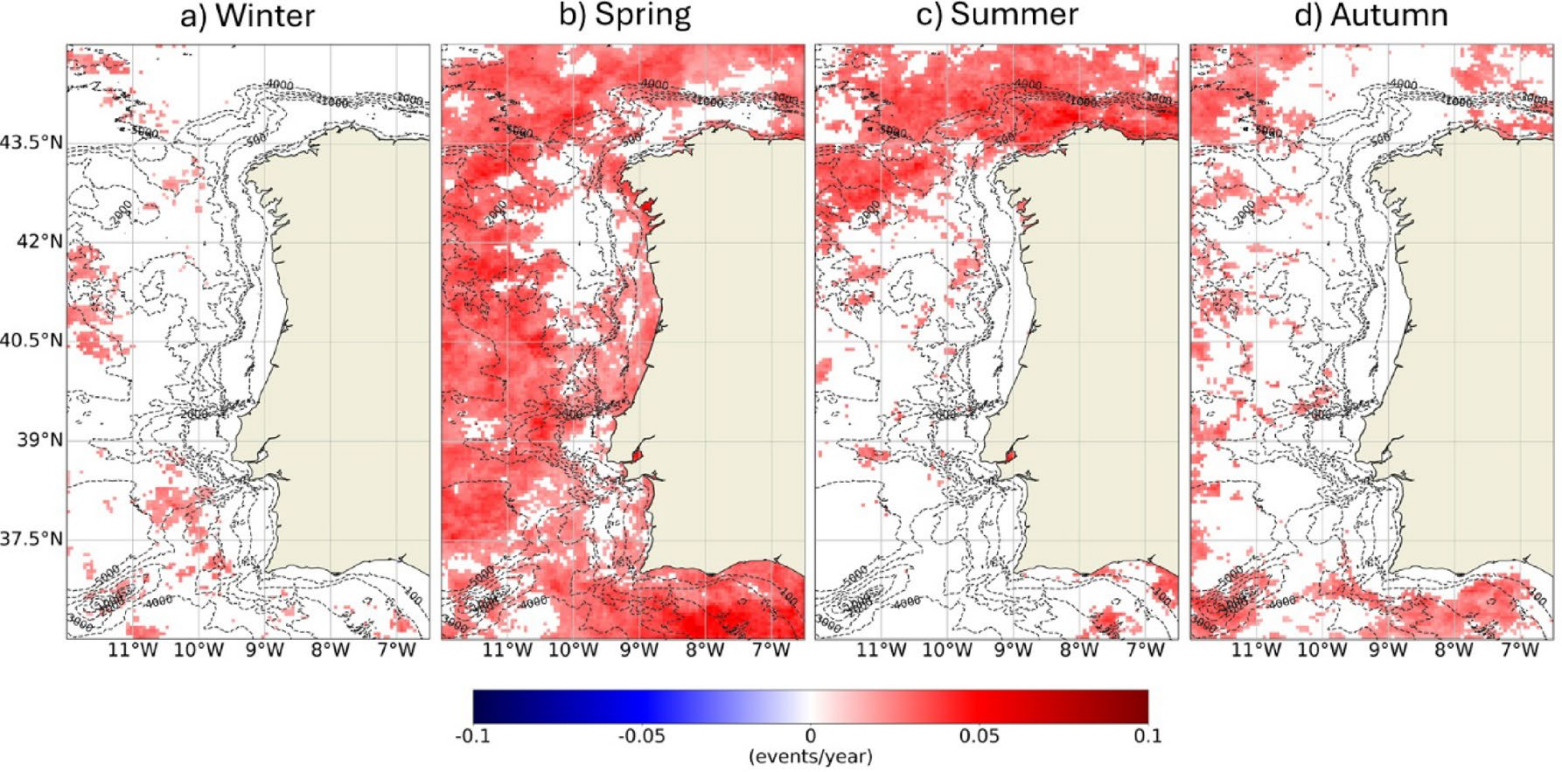
Trends (change/year) of the number of MHWs observed between 1982 and 2022 according to the season of the year in which they occurred. Only significant changes are colored (p-value < 0.05). Results obtained using the satellite-derived Sea Surface Temperature data from ESA CCI project. Bathymetry data source: GEBCO Compilation Group (2022) GEBCO 2022 Grid (doi: 10.5285/e0f0bb80-ab44-2739-e053-6c86abc0289c).

The changes observed regarding the first day of the events and their duration were overall very scattered and not significant. Along the west shore of the WIC, there were no significant changes in the frequency of events. However, along the North and Southern margins and offshore, the number of events has increased significantly by around 0.07 events/year, which translates into an increase of almost 3 events after 41 years. Along the west coast, the transition from increase to no trend appears to be related to the bathymetric features of the region, more specifically the transition from the slope to the continental shelf. Figure 9 shows the trends in the number of events according to the season of the year and highlights that the most widespread increase in the number of MHWs has been observed during spring, both offshore and along the continental shelf. The north of the region has also seen a significant increase on the number of MHWs during summer months. There were no other relevant significant changes derived from the seasonal analysis of the trends of the remaining parameters.

**Fig. 10:**
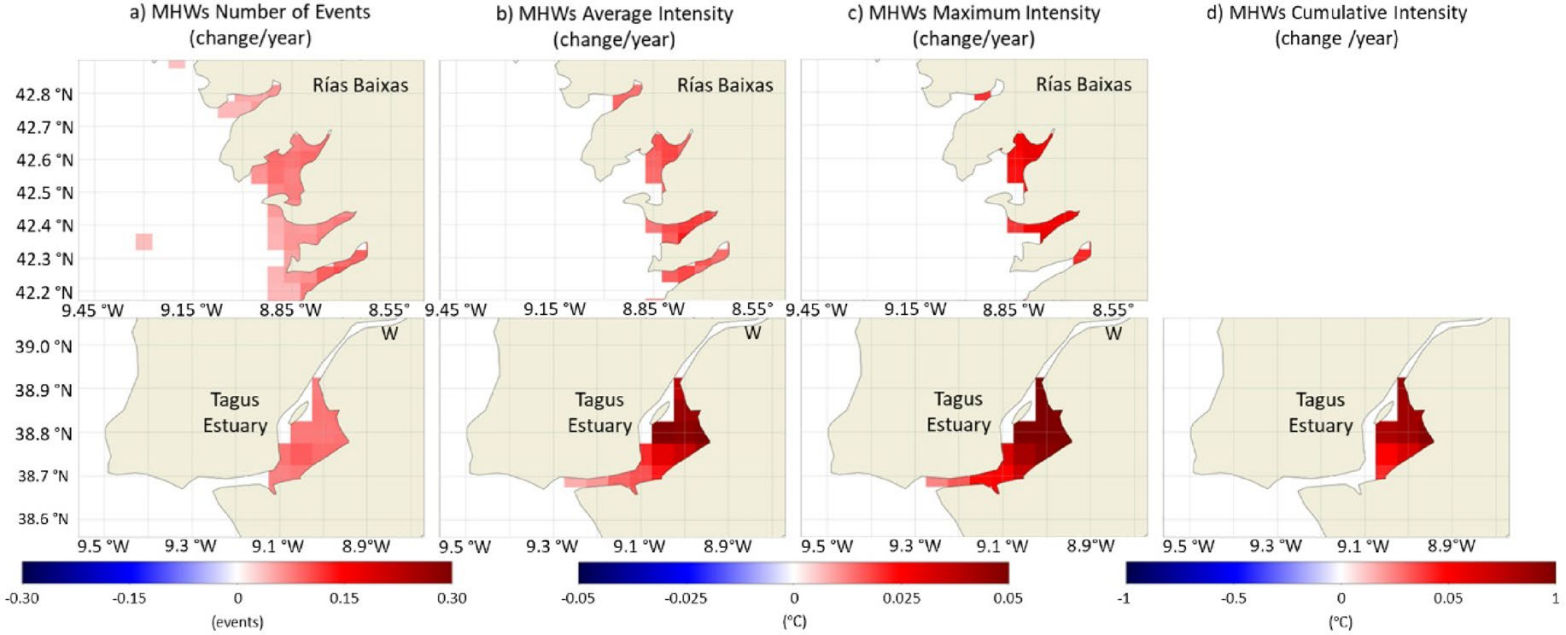
Trends (change/year) of the number of MHWs per year (**a**) and of their average (**b**), maximum (**c**) and cumulative intensity (**d**), along Rías Baixas and the Tagus Estuary, considering the period between 1982 and 2022. Only significant changes are colored (p-value < 0.05). Results obtained using the satellite-derived Sea Surface Temperature data from ESA CCI project.

In summary, no big significant changes were observed throughout the WIC. However, some estuarine systems were relevant exceptions and registered distinct significant changes, namely the Tagus Estuary and Rías Baixas (Fig. 10). The Tagus Estuary was the area of the studied region that presented the strongest trends towards intensification of the average and maximum intensity of MHWs. This increase was ≈ 0.06 ℃/year; over the 41-year period, the average and maximum intensity of MHWs increased around 2.5 ℃ in Tagus Estuary. Additionally, this was the only system where a significant increase in cumulative intensity was recorded. With similar patterns, although with weaker trends, an increase in the average and maximum intensity of the MHWs was observed in the Rías Baixas (≈ 0.014–0.028 ℃/year). In both systems, the number of MHWs has increased significantly, contrary to what was observed in the adjacent coastal ocean. This increase derives from a restricted increasing frequency of events starting during spring and summer (Fig. 9).

**Fig. 11:**
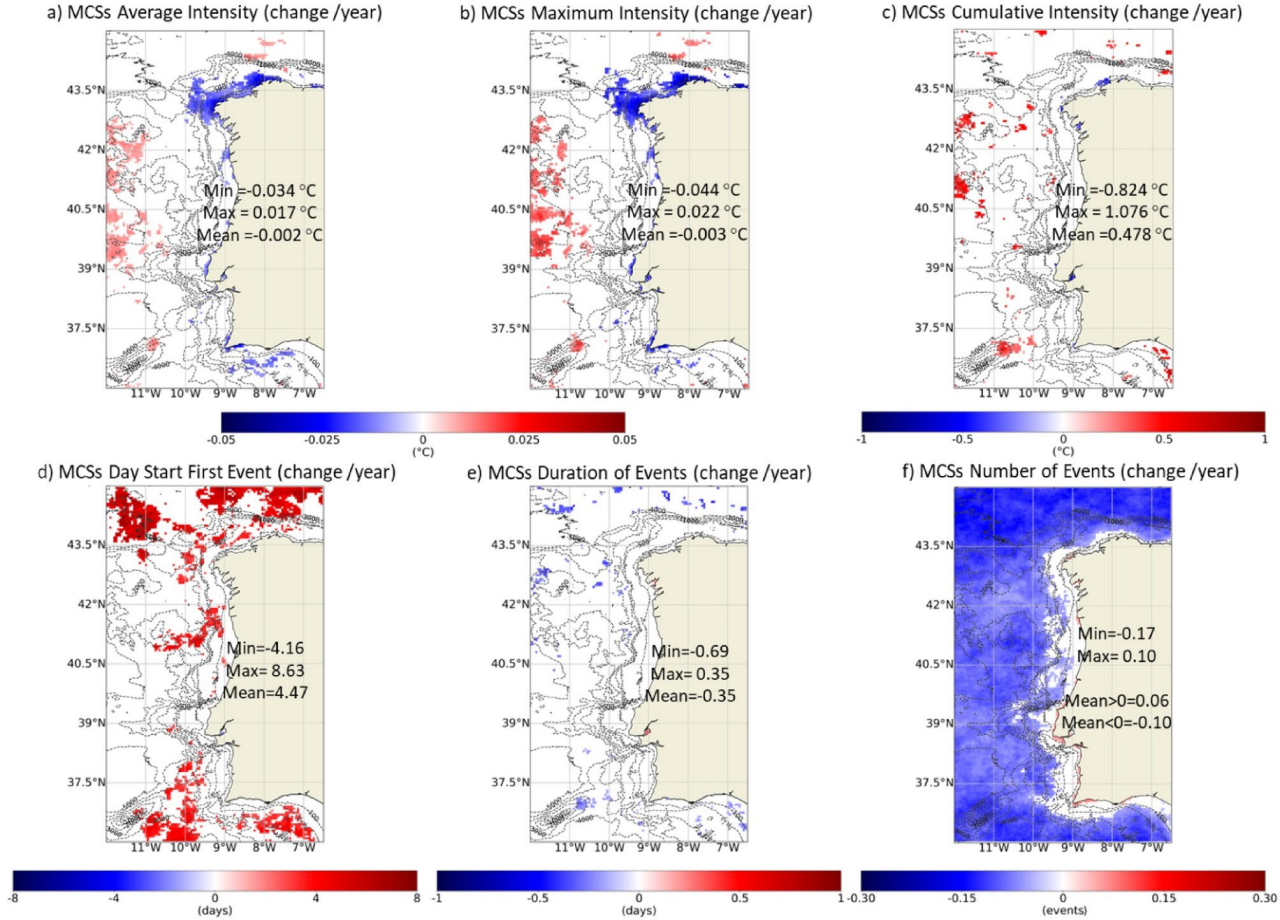
Trends (change/year) of MCSs average (**a**), maximum (**b**) and cumulative intensity (**c**), first day of the event (**d**), duration (**e**) and number of events per year (**f**) considering the period between 1982 and 2022. Only significant changes are colored (p-value < 0.05). Results obtained using the satellite-derived Sea Surface Temperature data from ESA CCI project. Bathymetry data source: GEBCO Compilation Group (2022) GEBCO 2022 Grid (doi: 10.5285/e0f0bb80-ab44-2739-e053-6c86abc0289c).

**Fig. 12:**
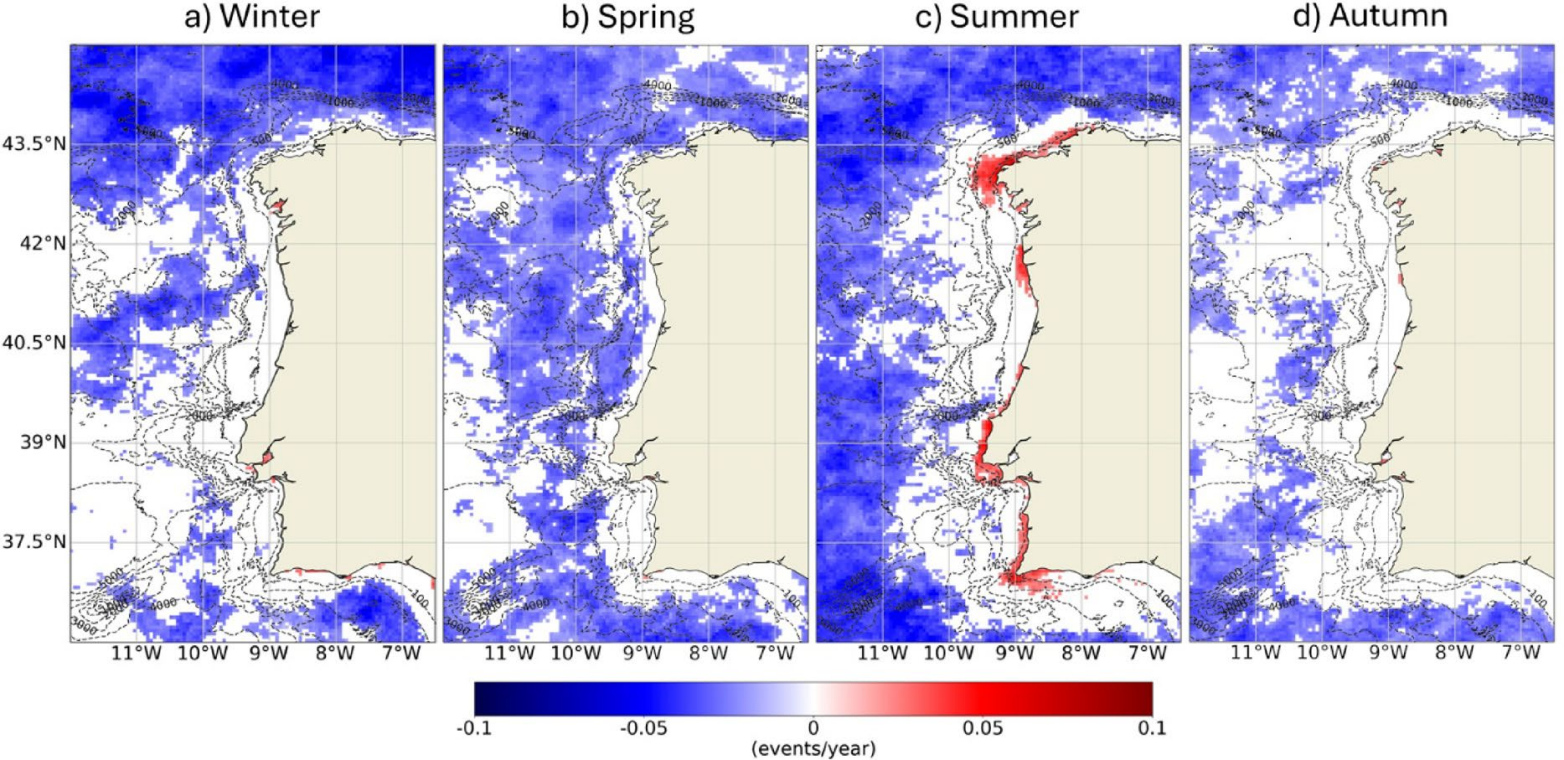
Trends (change/year) of the number of MCSs observed between 1982 and 2022 according to the season of the year in which they occurred. Only significant changes are colored (p-value < 0.05). Results obtained using the satellite-derived Sea Surface Temperature data from ESA CCI project. Bathymetry data source: GEBCO Compilation Group (2022) GEBCO 2022 Grid (doi: 10.5285/e0f0bb80-ab44-2739-e053-6c86abc0289c).

The equivalent analysis was done for MCSs and it is shown in Fig. 11, which follows the structure of Fig. 8 (average (a), maximum (b) and cumulative intensity (c), first day of the event (d), duration (e) and number of MCSs per year (f)). Once more, it should be noted that higher intensities correspond to lower values (negative values). Occasionally (e.g., in Cape São Vicente), an increase in the average and maximum intensity of MCSs was observed near the coast, with greater spatial relevance near Galicia (≈ 0.03–0.04 ℃/year). No significant changes were observed near the coast in the cumulative intensity, the first day of MCSs or the duration of events. MCSs started later in the year along some scattered offshore regions (average delay of 4.5 days/year). Regarding the frequency of MCSs, as with MHWs, the coastal region did not show significant changes. However, a significant decrease in the number of events was observed at offshore locations (average of − 0.1 events/year; less 4.1 events/year in 41 years). Near the coastline, positive trends were observed in the order of 0.06 events/year (2.5 events in 41 years). This pattern is only significant in the first few km of coast and presents a continuity almost from the Mondego Estuary to Ria Formosa. Once more, local bathymetric features could be influencing MCSs trends patterns. Figure 12 shows the trends of the number of MCSs according to the season of the year. The significant increase of the number of events observed along the first few km of coast for the whole period is similarly evident if only summer months are considered, as a significant increase in frequency of MCSs is observed, which is also very clear inside the Sado Estuary.

**Fig. 13:**
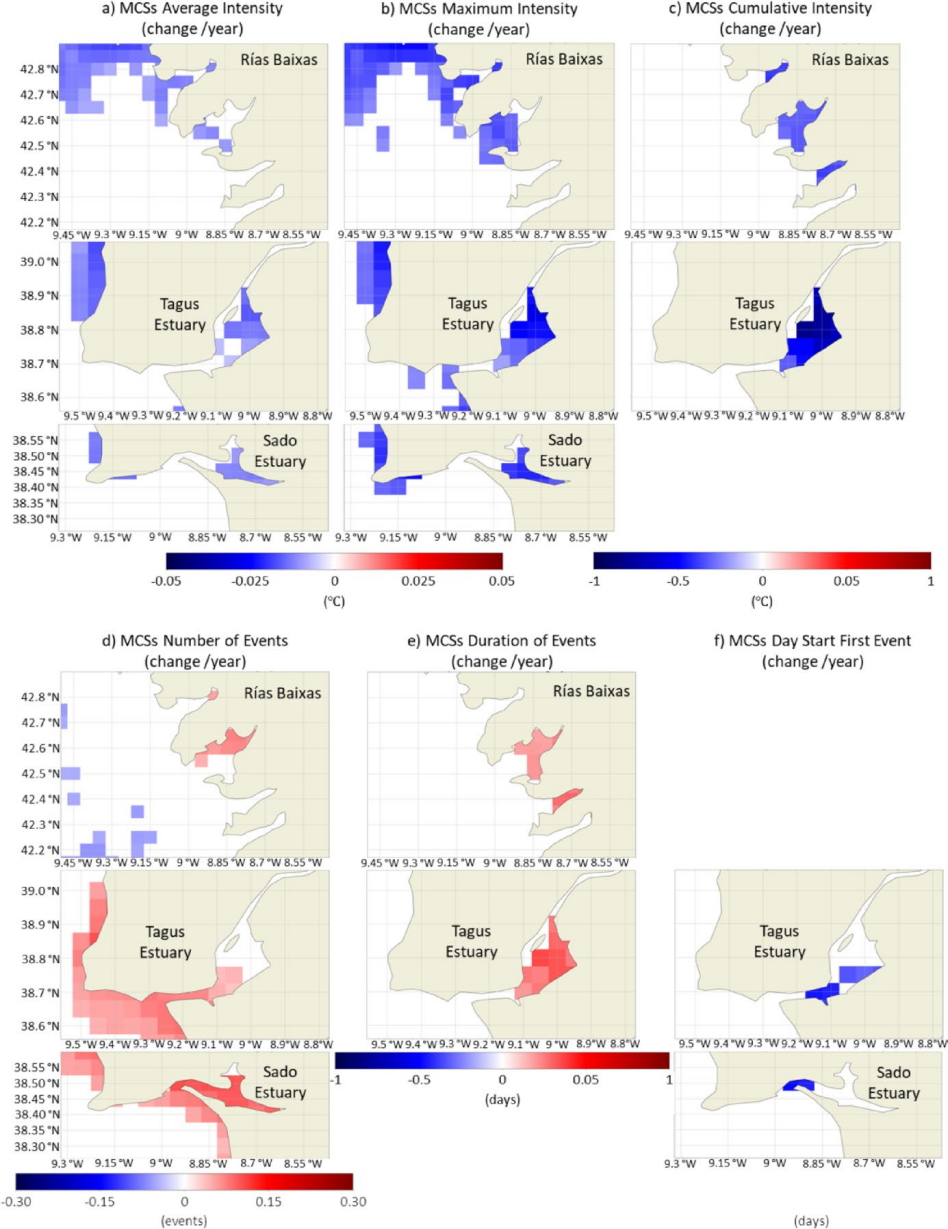
Trends (change/year) of the average (**a**), maximum (**b**) and cumulative intensity (**c**) of MCSs, the number of events per year (**d**), their duration (**e**) and the starting day of the first event (**f**), along Rías Baixas, Tagus and Sado Estuary, considering the period between 1982 and 2022. Only significant changes are colored (p-value < 0.05). Results obtained using the satellite-derived Sea Surface Temperature data from ESA CCI project

Some estuarine systems, namely Rías Baixas, Tagus Estuary and Sado Estuary, showed higher susceptibility to changes in MCSs features than the adjacent coast (Fig. 13). In Tagus and Sado Estuary and Rías Baixas, particularly in Rías de Muros-Noia and Arousa, an increase in the average and maximum intensity of events was observed, which was not significant in the adjacent coastal region. The same occurred inside the Mondego and Mira estuaries (only for maximum intensity in Mira), even though these systems are small relative to the spatial resolution of the product. The Sado Estuary in particularly, showed an overall increase of the mean and maximum intensity during winter, spring, and summer, in opposition to the remaining systems (data not shown). The highest trends in cumulative intensity were detected in the interior of the Tagus Estuary (− 0.8 ℃/year), with a clear significant incidence during summer, that was not detected during the other seasons (data not shown). These 3 systems, Rías Baixas, Tagus Estuary and Sado Estuary, also showed a significant increase in the number of MCSs, which was particularly relevant in the interior of the Sado Estuary, close to 0.1 events/year (more ≈ 4.1 events per year after 41 years). The results also point to an increase in the number of events in the interior of Minho, Lima, Douro, Mondego and Mira estuaries, as well as in small Rías from Rías Altas, near Finisterre. In the Tagus Estuary, the number of MCSs staring during winter and autumn has been significantly increasing, in opposition to the remaining time of the year (Fig. 12). Throughout the WIC, significant changes in the duration of MCSs were only observed in the Rías de Arousa and Pontevedra of Rías Baixas (that seasonally was significant for autumn; data not shown) and in the interior of the Tagus Estuary, which had an increase in duration of around 0.35 days/year. Although very localized, MCSs have been starting earlier in the year at a rate of ≈ 0.5 days/year at the mouth of the Sado Estuary and in certain inner areas of Tagus. Tagus Estuary is the system that has undergone the most significant changes both in MHWs and MCSs, revealing a particularly high susceptibility regarding these types of water temperature-related extreme events.

## Discussion

### Identification and classification

The highest intensity MHW detected along the WIC was Severe (Category III), lasted 61 days along the region of Cape São Vicente in the spring of 2011 and had a maximum intensity of 4.88 ℃. In the same year, Western Australia experienced an Extreme (Category IV) MHW of similar duration and intensity (4.89 ℃) but with dramatic biological impacts, namely on kelp forests, seagrasses, corals and economically important invertebrates^5^. Similarly, the highest intensity MCS identified in this study lasted 14 days and occurred offshore of the Mondego Estuary during the summer of 2002, reaching a maximum intensity of − 4.04 ℃ (Strong event). This temperature anomaly was like the Florida 2003 MCS, an event that lasted 40 days and promoted the development of phytoplankton blooms, the mortality of reef fish, interfered with fisheries and aggravated existing difficulties for recreational businesses^9^. To our knowledge, both events (Cape São Vicente 2011 MHW and Mondego Coast 2002 MCS) were first identified herein. Thus, no discussion on their possible effects on the coastal ecosystems have been provided yet.

The maximum intensity MHWs observed along the Portuguese west and south coastal regions corresponded to particularly hot summers, in which the average air temperature deviated more than 1.4 ℃ from the normal^31^. Higher air temperatures could be at the origin of these more intense events. However, only one Severe MHW was recorded in the past 40 years, suggesting the WIC is less susceptible to abnormal MHWs than other regions, such the Southwestern Patagonia, where 15 Category III and 1 Category IV events were identified between 1982 and 2020^32^. In the global assessment conducted by^8^, only Strong events were observed along the WIC, although severe MHWs may have been detected along southern region, which is partially in accordance with the results obtained here. Although the WIC is relevant for MCSs, only moderate and strong events were identified. The Southwestern Patagonia registered 158 MCS between 1982 and 2020, 15 of Category III and 6 of Category IV^32^. Even though the WIC registered a slightly higher incidence of MCSs, the absence of higher category events may reflect normal patterns. The WIC is located in an Eastern Boundary Upwelling Systems (EBUS) and it is influenced by coastal upwelling mostly between spring and summer. Wind-driven upwelling can boost MCSs and these may help marine ecosystem to recover when damaged by MHWs, as well as to buffer the impacts of heat stress during those type of extreme events^9,10^. Seasonally, both MHWs and MCSs peaked in summer (Fig. 5 and Figure S2). Along the southern coast, MCS events are more evenly distributed throughout the year, consistent with the region’s upwelling patterns^33^, further supporting the role of upwelling in MCSs development. Seasonal coastal upwelling could therefore explain why the WIC coast is not exceptionally favorable for the occurrence of both Severe and Extreme MHW and MCSs: it can buffer MHWs and enhance MCSs as part of the normal coastal patterns during summertime.

Very recently, a paper studied trends and variability of MHWs in Portuguese coastal waters^34^. Although the spatial resolution was lower, the authors documented more events in the south areas of the region, which agrees with the current assessment. Regarding MCSs, the information available for the region is very scarce. The western boundary currents (WBC) and the eastern equatorial Pacific are regions with higher incidence of MCSs^9^. Interestingly, WBC regions are also prominent in MHW frequency^35^. Here, the coastal regions from Minho to Douro and Cape São Vicente were also identified as most prone for both MHWs and MCSs. These regions have been identified as particular areas of upwelling development^36^. The south coast upwelling is mainly limited to Cape São Vicente. The higher incidence of upwelling development in these areas could justify why these were favorable for MCSs development. So, upwelling, which local characteristics are dependent on the bathymetry, additionally shapes the areas of higher incidence of MCSs. Eastern South Pacific MHWs are mainly associated to diminished heat loss and enhanced insolation, the result from a combination of positive (seaward) anomalies of air-sea heat fluxes and heat advection or driven by heat advection^37^. The positive phase of the Southern Annular Mode and the El Niño-Southern Oscillation (ENSO) were identified previously as primary drivers of increased MHW occurrence along the Northwest and Southwest coast of the WIC, along with the Dipole Mode index^35^. However, this analysis lacks regional resolution so further research on the topic should also be considered in the future. Either way, the WIC is not particularly favorable for the occurrence of MHWs, as also observed by^5^.

In the future, it would be important to understand if these events had any effects on the coastal systems (e.g., changes in species abundance and distribution, commercial fish stocks, and other ecosystem-services). For this it could be useful to consult the OCLE observatory (https://ocle.ihcantabria.com) that integrates meteo-oceanographic data and presences of marine species in a database of European coastal areas, where the WIC is also included. This database brings together various environmental variables such as wave height, wind speed, currents, sea level, sea and air temperature and salinity, among others, with an annual frequency between 2000 and 2022. Moreover, it would be very relevant to implement a national program for the identification and real-time monitoring of both MHWs and MCSs along the WIC and its estuarine systems, by providing daily updates and historical information on MHW/MCSs has already observed in other regions (e.g., Mediterranean Sea, with the Copernicus Mediterranean Heat Waves Monitoring Service). To name the events with higher intensity/higher classification would also be significant, as it would allow clarity when discussing historical events and enhance general public engagement by building awareness^5^.

### Average patterns

In a global analysis^38^, compared mean MCSs and MHWs statistics. The authors were able to map mean maximum intensities of MCS, and despite the relatively low spatial resolution, values were higher near the WIC than along the Atlantic coast. Also, they showed that this region registered on average a higher annual number of MCSs than the Atlantic and, simultaneously, more MCSs than MHWs^38^. Both the patterns and the magnitude of the mean maximum intensity obtained by these authors coincide with the results observed here (between 2 and 3 °C, higher near the WIC than offshore). A global assessment conducted by^39^ that focused solely on MHWs, also allowed to detect that the WIC was not characterized by a particular higher incidence of MHWs, despite the higher mean intensities observed near coastal regions (2.0–2.5 ℃). Also, despite lacking detailed regional coverage, it is seen that MHWs lasted on average between 10 and 15 days throughout the WIC. These results are in line with those obtained herein. Similar patterns were obtained by^40^ in a distinct global analysis. The regional study conducted by^34^ for the Portuguese coast also shows similar patterns and range of values for intensity and duration of MHWs. All the quoted papers show that the average coastal patterns for the WIC are similar to those observed along some areas of northwest Africa and California, even if with a smaller spatial extension. The Mediterranean Sea, despite the geographical proximity, had on average longer-lasting MHWs with higher mean and cumulative intensities^13^.

In the present study, for both MCSs and MHWs, the coastal regions between Minho and Aveiro and the south coast of Portugal had the highest average and maximum intensities. Moreover, the longest MHWs were on average recorded in SW region of Portugal, where the shortest MCSs were identified. The regional differences in the intensity and duration of the events could derive from the effects of offshore oceanographic processes, including the advection of anomalies by major currents, the activity of mesoscale eddies and meanders, and variability in upwelling and downwelling regime, as well as the local characteristics of the coast and its bathymetry^41^. Moreover, they can be attributed to differences in SST variance^9,42^. The variance of SST, representing SST spread in absolute terms, was estimated across the WIC from 1982 to 2022 and is shown in Supplementary Figure S3. The spatial variability of the SST variance near the coast is relatively small, so a qualitative color scale was chosen to obtain a better spatial perception of this variation. It appears that the areas with MHWs of greatest cumulative intensity (intensity cumulated over the duration of the event) overlap with areas of slightly lesser SST variance, while for the MCS, the areas with events of higher cumulative intensity appear to correspond to areas of greatest SST variance^42^. also identified that the maximum intensity of MHWs and MCSs was greatest along the same regions of the Southern North Sea, i.e., the northern Dutch coast and in the German Bight. The authors concluded that this regional correspondence between the two types of events arises from a higher SST variability in those locations. In this case, the opposite is observed.

### Trend analysis

Despite global evidence, the WIC has not been suffering significant changes regarding MHWs features. The upwelling of colder sub-surface waters resulting from a seasonal predominance of northerly winds makes this and other similar regions to act as buffers for water temperature increases as a consequence of global warming^12^. The WIC was previously identified as having regions that work as a buffer for SST changes due to upwelling and no significant SST trends were identified near-shore between 1982 and 2021^29^. In fact^43^, detected lower trends in the number of MHWs days near the coast than in the adjacent ocean unaffected by upwelling events in the Benguela, Canary, Chile, Peru and California EBUs systems, suggesting that upwelling can moderate the occurrence of MHWs compared with the adjacent ocean. Significant changes in MHWs intensity were detected only near the south shore of the WIC (Algarve). The frequency of upwelling occurrence in the south coast tends to be 50% of the upwelling occurrences in the west coast, which means that this region is much less influenced by this phenomenon^33^. Despite the overall lack of significant changes along the WIC, caution should be taken with future assessments, as other EBUSs, such as those in the Southern Hemisphere, have been detected as future local MHWs hotspots^12^.

In opposition, MCSs incidence has been decreasing worldwide, along with their intensity^12,41^. Along the Northeast Atlantic, a decrease in MCS activity was detected with almost no events after 2000^16^. Significant warming trends are a straightforward explanation for the general weakening found in MCS metrics^9^. The Southern Ocean region and eastern equatorial Pacific are known exceptions^9^. Along the WIC, only local changes in MCSs physical attributes were significant. Although an offshore decrease in the occurrence of MCSs was observed, no significant changes were observed along the WIC and an increase was registered near the coastline, that has been significantly increasing over the summers of the past 4 decades (Fig. 12). Beyond the buffer effect^29^, detected significant SST decreasing trends near the WIC shore based on daily SSTs and attributed these patterns in part to a possible intensification of upwelling events. Seasonal upwelling local characteristics are dependent on the bathymetry, and therefore this phenomenon could be once more playing an important role in defining both MHWs and MCSs trend patterns^9^. mentions that regions of the ocean where MCSs are not diminishing tend also to be regions where MHWs are not increasing, as here observed along the near-shore WIC. Supplementary Figure S4 illustrates the previous points: it visually compares the observed trends in the number of MHWs and MCSs along the WIC with SST annual trends and the correlation between SST annual anomalies with Upwelling Index. It presents a clear link between SST and upwelling long-term spatial patterns to the observed distributions of MHWs and MCSs trends, highlighting spatial differences from near shore to offshore, which increases the confidence in this discussion.

Some WIC estuarine systems, however, showed a particularly high susceptibility to changes in MHWs and MCSs proprieties. Changes in global climate places shallow water ecosystems at more risk as their SST could change more rapidly and dramatically^41^. Despite the potential higher susceptibility, there are very few studies focusing on MHWs and MCSs attributes in such systems. Along the WIC, there were significant changes in more estuarine systems when considering MCSs than when considering MHWs. This means that, in general, the dynamics of estuarine systems in the WIC could be more influenced by MCS.

The Tagus Estuary and Rías Baixas revealed a particularly high susceptibility to long-term changes in the MHWs. This greater susceptibility could derive from the significant increase in SST observed since 1982^29^. This trend was not clearly observed in other WIC estuaries and its magnitude was equivalent to that in the coastal ocean, not corroborating the patterns observed along the coastline. The Tagus Estuary stood out for presenting the highest increase in SST. These were also the systems that presented a higher susceptibility to long-term changes in the characteristics of MCSs, particularly the Tagus Estuary. The Sado Estuary proved to be one of the most prone regions for MCSs development and significant trends, but this system has seen an exceptional (and significant) decrease in SST since 1982^29^, which has been associated to a decrease in the Sado river flow, changes of wind patterns and a higher influence of upwelled waters inside of the estuary^44^. The system appears to have a buffering role for the increase in temperature which could be related to the higher occurrence of MCSs. The variability in the Tagus Estuary is influenced by coastal process, namely upwelling^45^ but it is not clear how upwelling conditions have been changing in this region. Further studies on this topic should be conducted to effectively conclude on the reasons behind the higher susceptibility of this estuary to MCSs changes.

## Conclusions

In this paper, we compared past MHWs and MCSs along the WIC and its estuarine systems in terms of their patterns and physical attributes, for the first time to our knowledge. The first objective of this study was to identify past events, classify them and determine the most favorable regions to their occurrence. On average, there were more MCSs than MHWs over the WIC. Only one Category III MHW was identified between 1982 and 2022 and no MCSs exceeding Category II intensities were detected. The highest intensity MHWs and MCSs were observed during summer. The WIC coast was not exceptionally favorable for the occurrence of MHWs. In opposition, there was a clear higher incidence of MCSs near the coast than offshore. The coast between Minho and Douro Estuaries and Cape São Vicente were identified as most prone for the occurrence of both MHWs and MCSs. Rías Baixas and the Sado Estuary proved to be more favorable for MCSs development.

The second objective of this study was to evaluate the average patterns of the last 41 years regarding MHWs/MCSs physical attributes. The coast between Minho and Aveiro and the south coast of Portugal had the highest average and maximum intensity MHWs and MCSs. On average, the longest MHWs (≈ 17 days) were recorded in SW region of Portugal, where the shortest MCSs were identified (≈ 10 days). The third and last objective of this study was to conduct a trend analysis. No MHWs significant changes were observed throughout the west coast of the WIC. A significant decrease in the number of MCSs was observed offshore (average of − 0.1 events/year), while an increase was registered near the coastline (0.06 events/year). The Tagus Estuary revealed a higher susceptibility to changes in the physical attributes of both MHWs and MCSs. The seasonal upwelling along the WIC could explain the observed results.

Our results are a relevant contribution to the development of adaptation and mitigation approaches against the effects of extreme water temperature events. Furthermore, the recognized lack of scientific evidence needed to support various interdisciplinary climate change research proposals focused on the WIC and its estuaries will be overcome.

## Supplementary Information

Below is the link to the electronic supplementary material.

Supplementary Material 1

## Data Availability

The datasets generated and/or analyzed during the current study are available in the Copernicus Climate Data Store repository, https://cds.climate.copernicus.eu/datasets/satellite-sea-surface-temperature? tab=overview.
