# Supplementary material for: Features and trends of marine heat waves and marine cold spells along the Western Iberian Coast from four decades of satellite data
Source: Sci Rep. 2025 Dec 24;16:1860. doi: 10.1038/s41598-025-31504-1 (PMC12804181; doi:10.1038/s41598-025-31504-1)
Supplement: Supplementary file 1 — Supplementary Material 1 [file 41598_2025_31504_MOESM1_ESM.docx]

Supplementary Materials

Features and Trends of Marine Heat Waves and Marine Cold Spells Along the Western Iberian Coast from Four Decades of Satellite Data

Beatriz Biguino^1,*^, Ivan D. Haigh^2^, João Miguel Dias^3^, Ana C. Brito^1,4^

^1^ MARE - Marine and Environmental Sciences Centre / ARNET - Aquatic Research Network, Faculdade de Ciências, Universidade de Lisboa, 1749-016 Lisboa, Portugal;

^2^ School of Ocean and Earth Science, National Oceanography Centre, University of Southampton, Waterfront Campus, European Way, Southampton SO14 3ZH, UK.

^3^ Centre for Environmental and Marine Studies (CESAM), Departamento de Física, Universidade de Aveiro, 3810-193 Aveiro, Portugal

^4^ Departamento de Biologia, Faculdade de Ciências, Universidade de Lisboa, 1749-016 Lisboa, Portugal

^*^ email: bibiguino@fc.ul.pt

Table S1. Variance and coefficient of variation of the number of MHWs and MCSs obtained considering the 3x3 pixel frame of each location (A-G). Variance was calculated following $\frac{\sum_{i} {|a_{i}-\bar{a}|}^{2}}{N}$, where $a_{i}$refers to each data point, $\bar{a}$ is the mean of all values and N is the number of elements. The coefficient of variation was calculated following $\frac{Standard Deviation}{Mean}$.

| MHWs | | | | | | | |
| --- | --- | --- | --- | --- | --- | --- | --- |
| Location | A | B | C | D | E | F | G |
| Variance | 3.951 | 13.580 | 11.506 | 7.877 | 8.691 | 4.395 | 16.395 |
| Coefficient of variation | 0.024 | 0.047 | 0.043 | 0.036 | 0.037 | 0.024 | 0.050 |
| MCSs | | | | | | | |
| Location | A | B | C | D | E | F | G |
| Variance | 38.173 | 11.728 | 9.284 | 17.778 | 7.778 | 16.173 | 4.840 |
| Coefficient of variation | 0.082 | 0.041 | 0.030 | 0.055 | 0.032 | 0.044 | 0.025 |


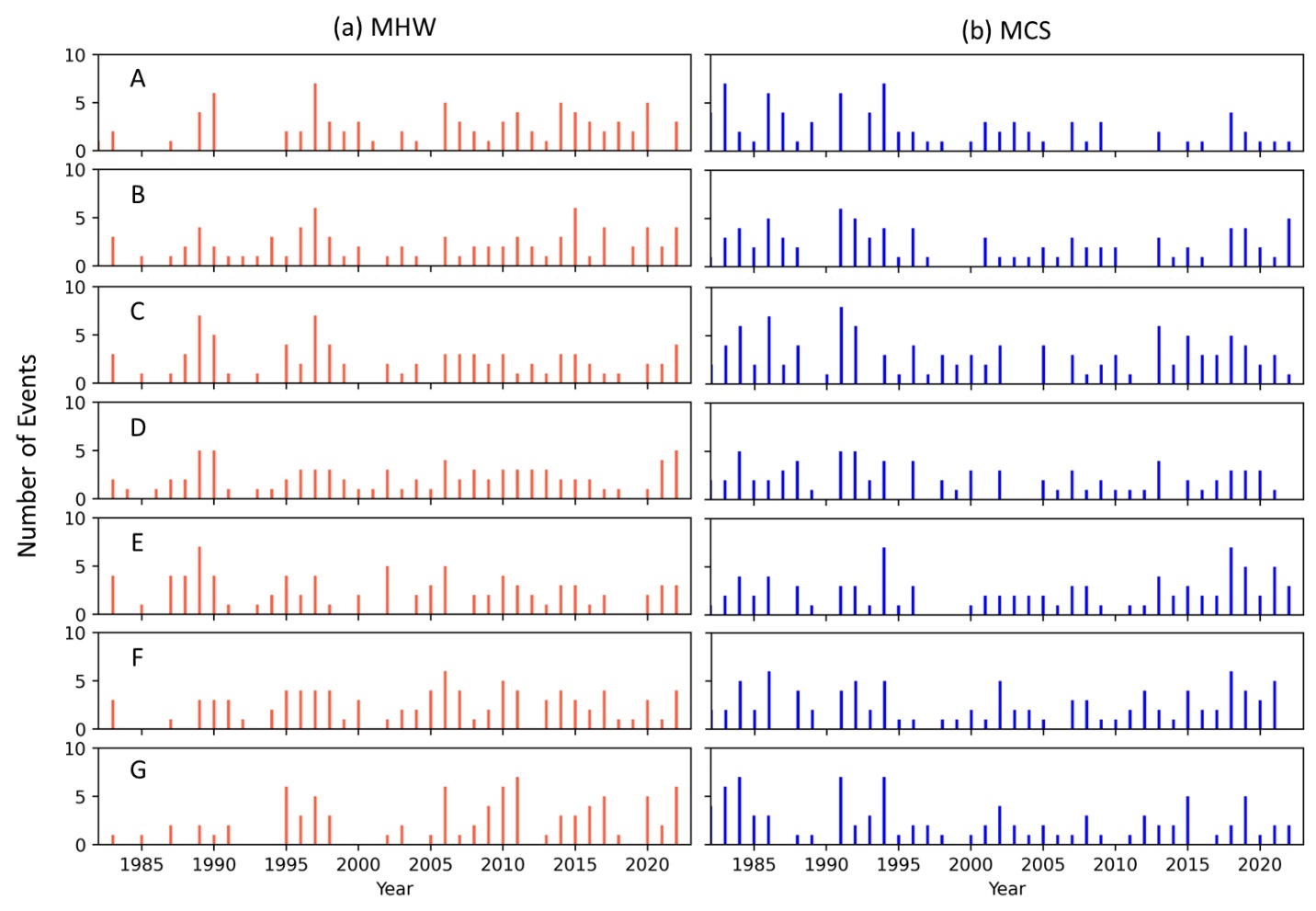


Figure S1. Number of Marine Heat Waves (a) and Marine Cold Spells (b) observed each year from 1982 to 2022 at the 7 studied locations of the Western Iberian Coast (A-G).


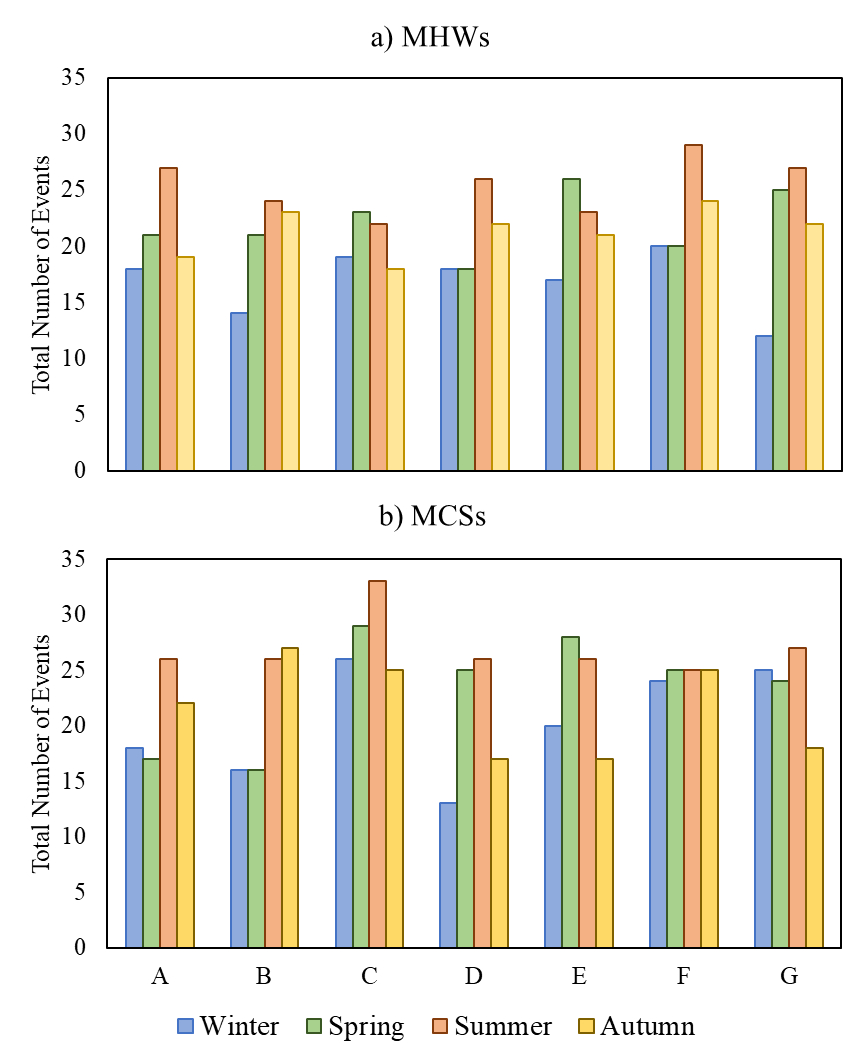


Figure S2. Total number of Marine Heat Waves (MHWs) and Marine Cold Spells (MCSs) obtained for the 7 studied regions of the Western Iberian Coast according to the season of the year. Winter: JAN-MAR; Spring: APR-JUN; Summer: JUL-SEP; Autumn: OCT-DEC.


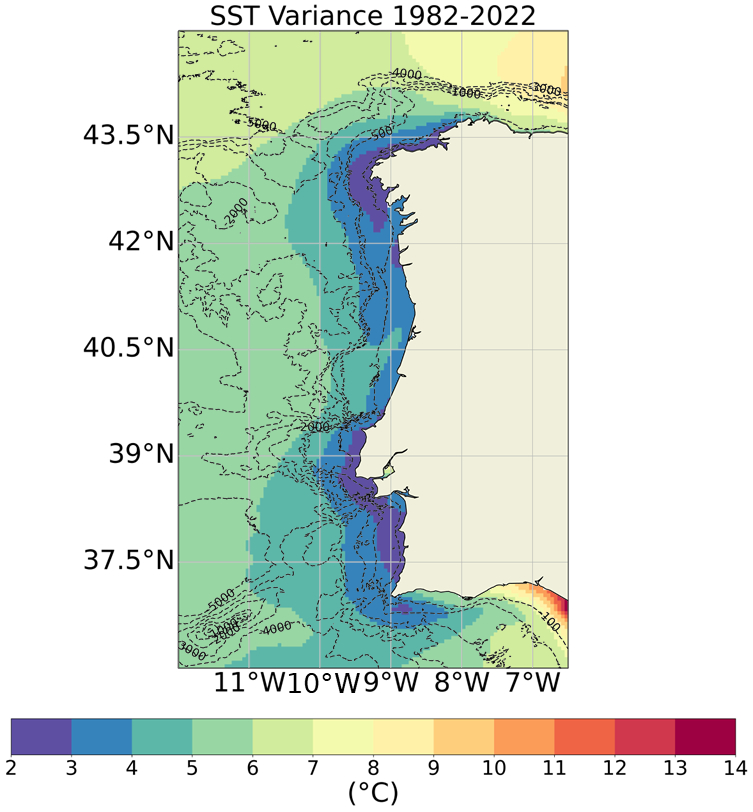


Figure S3. Variance of sea surface temperature between 1982 and 2022 (ºC). Variance was calculated as in Supplementary Table S1. Results obtained using the satellite-derived SST data from ESA CCI project. Bathymetry data source: GEBCO Compilation Group (2022) GEBCO 2022 Grid (doi: https://doi.org/10.5285/e0f0bb80-ab44-2739-e053-6c86abc0289c).


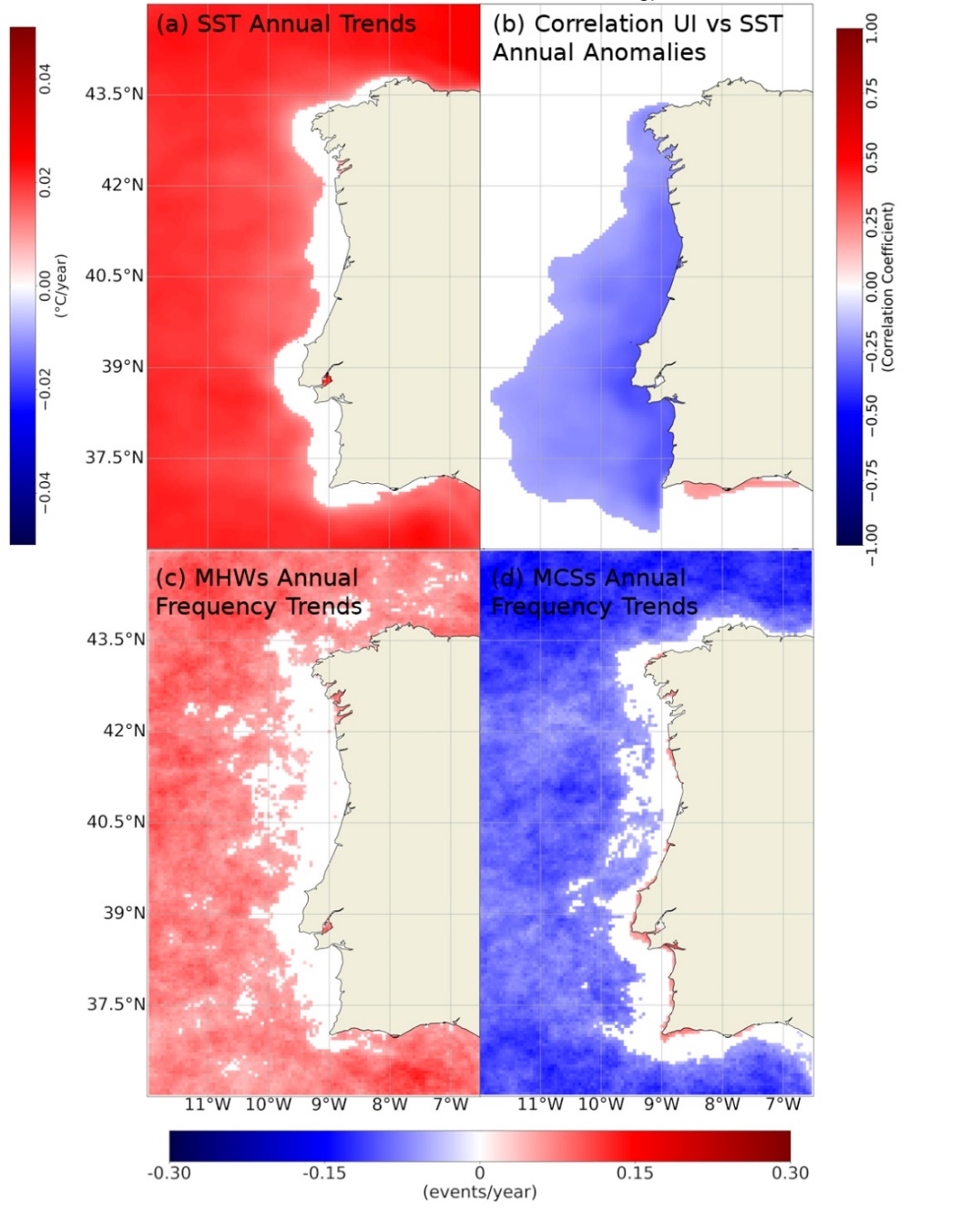


Figure S4. (a) Sea surface temperature annual trends in ºC/year (adapted from Biguino et al., 2021); (b) Correlation coefficient between sea surface temperature annual anomalies and annual values of Upwelling Index (from Biguino et al., 2021); (c) Marine Heat Waves (MHWs) annual frequency trends (events/year); (d) Marine cold Spells (MCSs) annual frequency trends (events/year). Results obtained using the satellite-derived SST data from ESA CCI project between 1982 and 2022. Only significant results are colored (p-value<0.05).
